# Identification of Potent and Selective JAK1 Lead Compounds Through Ligand-Based Drug Design Approaches

**DOI:** 10.3389/fphar.2022.837369

**Published:** 2022-04-21

**Authors:** Sathya Babu, Santhosh Kumar Nagarajan, Sruthy Sathish, Vir Singh Negi, Honglae Sohn, Thirumurthy Madhavan

**Affiliations:** ^1^ Computational Biology Lab, Department of Genetic Engineering, School of Bioengineering, SRM Institute of Science and Technology, SRM Nagar, Kattankulathur, India; ^2^ Department of Clinical Immunology, Jawaharlal Institute of Post-Graduate Medical Education and Research, Pondicherry, India; ^3^ Department of Chemistry and Department of Carbon Materials, Chosun University, Gwangju, South Korea

**Keywords:** JAK1, pharmacophore modeling, virtual screening, molecular docking, molecular dynamics simulation, density function theory

## Abstract

JAK1 plays a significant role in the intracellular signaling by interacting with cytokine receptors in different types of cells and is linked to the pathogenesis of various cancers and in the pathology of the immune system. In this study, ligand-based pharmacophore modeling combined with virtual screening and molecular docking methods was incorporated to identify the potent and selective lead compounds for JAK1. Initially, the ligand-based pharmacophore models were generated using a set of 52 JAK1 inhibitors named C-2 methyl/hydroxyethyl imidazopyrrolopyridines derivatives. Twenty-seven pharmacophore models with five and six pharmacophore features were generated and validated using potency and selectivity validation methods. During potency validation, the Guner-Henry score was calculated to check the accuracy of the generated models, whereas in selectivity validation, the pharmacophore models that are capable of identifying selective JAK1 inhibitors were evaluated. Based on the validation results, the best pharmacophore models ADHRRR, DDHRRR, DDRRR, DPRRR, DHRRR, ADRRR, DDHRR, and ADPRR were selected and taken for virtual screening against the Maybridge, Asinex, Chemdiv, Enamine, Lifechemicals, and Zinc database to identify the new molecules with novel scaffold that can bind to JAK1. A total of 4,265 hits were identified from screening and checked for acceptable drug-like properties. A total of 2,856 hits were selected after ADME predictions and taken for Glide molecular docking to assess the accurate binding modes of the lead candidates. Ninety molecules were shortlisted based on binding energy and H-bond interactions with the important residues of JAK1. The docking results were authenticated by calculating binding free energy for protein–ligand complexes using the MM-GBSA calculation and induced fit docking methods. Subsequently, the cross-docking approach was carried out to recognize the selective JAK1 lead compounds. Finally, top five lead compounds that were potent and selective against JAK1 were selected and validated using molecular dynamics simulation. Besides, the density functional theory study was also carried out for the selected leads. Through various computational studies, we observed good potency and selectivity of these lead compounds when compared with the drug ruxolitinib. Compounds such as T5923555 and T5923531 were found to be the best and can be further validated using *in vitro* and *in vivo* methods.

## Introduction

Janus kinase 1 (JAK1) is the most widely employed JAK, according to biochemical and genetic research, since it is involved in the signaling of the gamma common (γc), beta common (βc), gp130, type I and type II interferon, IL-6, and IL-10 cytokine subfamilies (Kulagowski et al., 2012). JAK1 comprises seven homology domains (JH1–JH7) (Harpur et al., 1992). The C-terminal kinase module (JH1) is the protein’s physiologically active catalytic domain. The JH2 domain is a catalytically inactive pseudokinase domain that has been found to interact with the JH1 domain and control its activity (Saharinen and Silvennoinen 2002). Two Src homology 2 (SH2) domains (JH3 and JH4) precede the FERM domain (JH5–JH7) at the N-terminus. The JH1 domain has an ATP-binding site, which has been the target of a number of small-molecule inhibitors. All four members of JAK have a highly conserved kinase domain, particularly at the ATP-binding region, which complicates the development of particular inhibitors (Caspers et al., 2016). The active sites of JAKs comprise multiple subdomains that include the β-glycine loop, the catalytic loop, and activation loops (Taldaev et al., 2022). The amino acid present in and around the hinge region serves critical functions in the integrity of kinase activity control. Furthermore, since this area is adjacent to the ATP-binding site in the catalytic cleft, it is reasonable to believe that the mutations in this region might promote constitutive activation of the kinase (Gorden et al., 2010; Haan et al., 2010).

All STAT proteins (STAT1–STAT6) that are ubiquitously expressed in all the tissues may be phosphorylated by JAK1 enzyme (Gruber et al., 2020). JAK1 has been shown in mouse knockout experiments to have a critical function in signal transduction (Itteboina et al., 2017). According to earlier research, JAK1 is ascendant over JAK3, and in the absence of JAK1, JAK3 is unable to activate STATs (Haan et al., 2011). Furthermore, recent studies have shown that JAK1 rather than JAK3 kinase is the primary driver of the immune-relevant cytokine activity (Menet et al., 2015). JAK1 is involved in various types of cancer. Activation of JAK1 kinase by IL-6 family cytokines appeared to be the mechanism for constitutive STAT3 activation in human ovarian cancer cells (Wen et al., 2014). In gastric cancer, by activating the JAK1/STAT3 pathway, the upregulation of HOXA10 gene increased cell proliferation, cloning formation, and tumorigenesis and lowered cell apoptosis (Chen et al., 2019). In lung adenocarcinoma patients, the overall survival time was substantially reduced in patients with EGFR-amplified tumors expressing greater levels of phosphorylated JAK1 compared with individuals with tumors without one or both of these traits. Additionally, JAK inhibition was demonstrated to limit the development of human lung adenocarcinoma with a K-RAS mutation (Xie et al., 2021). AML and breast cancer patients have exhibited several STAT5-activating JAK1 mutations (Hornakova et al., 2011). Moreover, in ER-negative breast cancer cell lines, the upregulation of phosphorylated JAK1 expression was observed (Yeh et al., 2007).

According to clinical and experimental investigations, rheumatoid arthritis synovial response may be influenced by the JAK1-mediated cytokine (IFN and IL-6) signaling. As a result, inhibiting JAK1 is regarded as a significant therapeutic strategy for the successful treatment of rheumatoid arthritis (Keretsu et al., 2021b). Recently, it has been discovered that inhibiting JAK1 selectively may be an effective therapy option for patients suffering from autoimmune and hematological illnesses because of the role that altered JAK1 signaling plays in these conditions (Kleppe et al., 2017). Moreover, JAK1 expression in cancer cells allows individual cells to contract, perhaps enabling them to transcend their tumor and spread to other areas of the body (Nordqvist 2011). Mutations in JAK1 are less common than in T-ALL patients with B-ALL or leukemia of the myeloid origin. In two AML patients, a JAK1 mutation V623A was found, emphasizing the capacity of constitutively active JAK1 to induce a variety of leukemias (Xiang et al., 2008; Raivola et al., 2021).

JAK inhibitors, which have been authorized for the treatment of cancer and autoimmune illnesses, have provided the first insight on the importance of JAK1 in NK cell biology (Schwartz et al., 2017). Ruxolitinib, JAK1/JAK2 inhibitor, has lowered the number of NK cells and hampered maturation and function in both mice and human patients (Schönberg et al., 2015; Bottos et al., 2016). Ruxolitinib’s influence on NK cell development has been linked to JAK2 as well; therefore, it is not clear which of the two kinases is accountable for the reported results (Bottos et al., 2016; Kim et al., 2017). The fascinating finding by Sohn et al. (2013) has highlighted the importance of JAK1 inhibitor on the IL-6, IL-22, and INF-pathways. JAK1 inhibitors including ruxolitinib, tofacitinib, filgotinib, peficitinib, and numerous additional second-generation inhibitors are now under investigation for the treatment of inflammatory and autoimmune illnesses. Because of limited potency, non-targeting, and off-target effects (Keretsu et al., 2021a), new JAK1 inhibitors with high potency and selectivity are urgently needed.

Pharmacophore models are widely employed to quantitatively explore common chemical characteristics among a considerable number of structures with great diversity (Taha et al., 2008; Xie et al., 2009). It is one of the widely used approaches to search for chemical databases and identify novel scaffolds for various targets (Wang H. et al., 2008; Lu et al., 2007). To discover the potent hits, the ligand-based and structure-based pharmacophore models can be used. In this study, the ligand-based pharmacophore models were generated using the 52 JAK1 inhibitors reported by Zak et al. It elucidates the spatial arrangement of structural features of various potent and structurally diverse inhibitors crucial for biological recognition. One efficacious approach toward the discovery and development of the drugs is the virtual screening of molecular libraries (Stahl et al., 2006). Virtual screening helps to identify the potential lead molecules and reduces the time and cost of the drug discovery process (Reddy et al., 2007). Thus, pharmacophore-based virtual screening was implemented. In many research works, it was proposed that the combination of pharmacophore modeling and molecular docking is a successful method to discover the novel and potent lead compounds (Sakkiah et al., 2009; Sakkiah et al., 2010; Sakkiah et al., 2011). Hence, the results of pharmacophore-based virtual screening were taken for molecular docking.

Docking results were used to predict the binding orientations of the hits as well as the filter to select the hits. The molecular docking results were validated by calculating the free energy of binding using the molecular mechanics-generalized born surface area (MM-GBSA) method for the protein–ligand complexes (Friesner et al., 2006). Furthermore, induced fit docking (IFD) was carried out to get additional understanding about the structure and flexibility of these hits into the binding site since IFD has been reported to be a powerful method to account for both receptor and ligand flexibility (Zhong et al., 2009). Subsequently, the cross-docking method was used to identify the selective hits by docking every hit to every receptor. By examining the results, the top five hits were selected and taken for molecular dynamic simulation and density functional theory (DFT) study. To identify the potency and selectivity of the leads, a drug molecule named ruxolitinib was included in the study. The results of selected lead compounds and the drug were compared and analyzed.

## Materials and Methods

### Dataset Selection

For ligand-based pharmacophore modeling, a set of 52 JAK1 inhibitors (C-2 methyl/hydroxyethyl imidazopyrrolopyridines derivatives) reported by Zak et al. (2012) and Zak et al. (2013) were selected because of their diverse biological activity. The K_i_ values of these inhibitors (0.1–150 nM) were derived using biochemical and cell-based assays. These inhibitors have shown higher selectivity toward JAK1 over JAK2. The experimental K_i_ values were converted into pK_i_ values that are simply the negative log of the K_i_ value. The chemical structures and biological activities of all molecules are given in Table 1.

**TABLE 1:** The chemical structures and the biological activity of JAK1 inhibitors.

Compound 1–21		Compound 22		Compound 23–32		Compound 33–53	
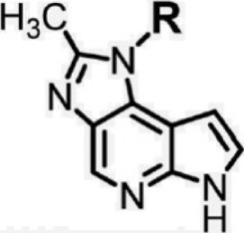		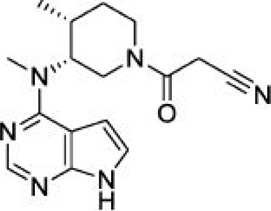		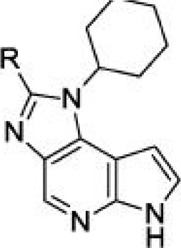		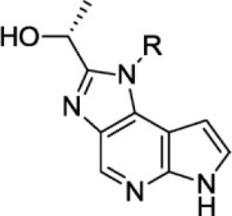	
**S. no.**	**R**	**K_i_ (nM)**	**pK_i_**	**S.no**	**R**	**K_i_ (nM)**	**pK_i_**
1	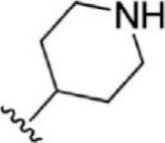	10	8.000	27	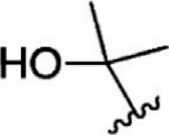	13	7.886
2	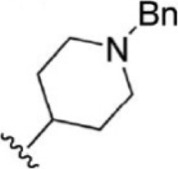	1.3	8.886	28	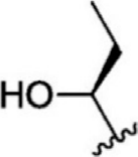	16	7.796
3	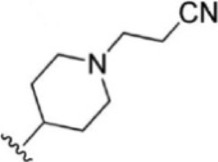	0.9	9.046	29	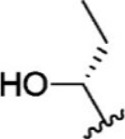	1.8	8.745
4	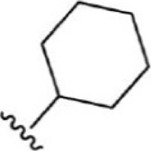	1.3	8.886	30	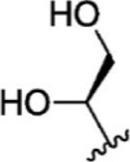	7.2	8.143
5	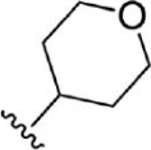	18	7.745	31	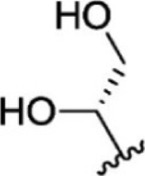	1.6	8.796
6	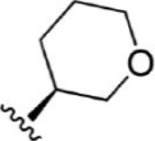	2.8	8.553	32	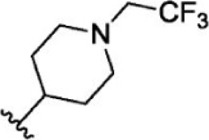	2.6	8.585
7	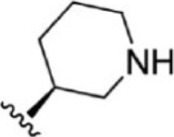	150	6.824	33	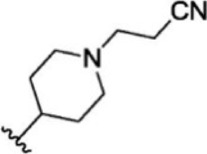	3.4	8.469
8	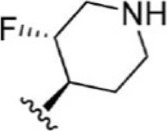	1.2	8.921	34	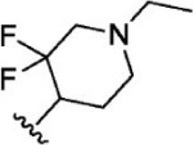	2	8.699
9	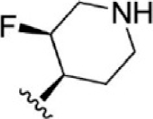	9.3	8.032	35	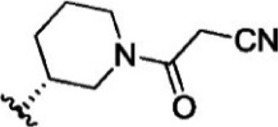	5.2	8.284
10	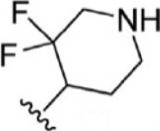	2	8.699	36	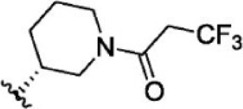	43	7.367
11	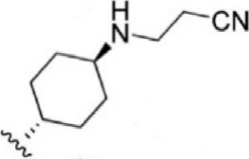	1.5	8.824	37	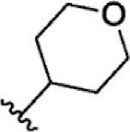	31	7.509
12	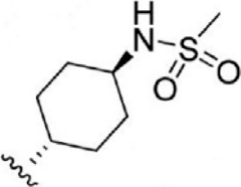	4.5	8.347	38	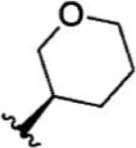	68	7.167
13	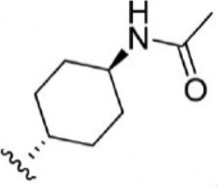	6.1	8.215	39	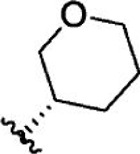	2.8	8.553
14	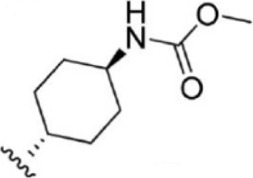	2.6	8.585	40	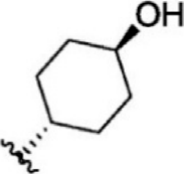	5.4	8.268
15	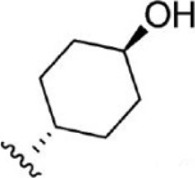	5.8	8.237	41	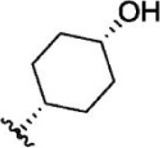	12	7.921
16	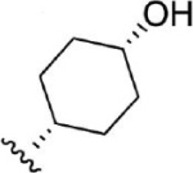	6.7	8.174	42	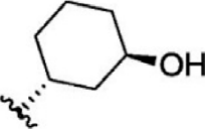	2.7	8.569
17	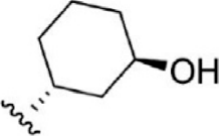	1.8	8.745	43	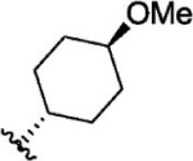	4.9	8.310
18	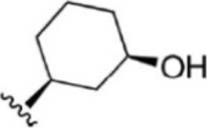	7.3	8.137	44	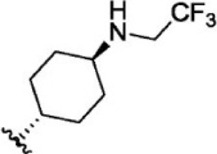	0.8	9.097
19	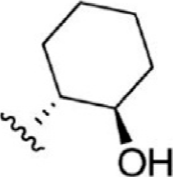	90	7.046	45	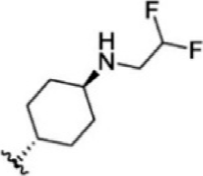	1.1	8.959
20	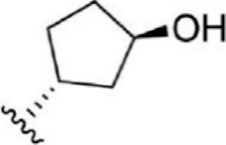	4.8	8.319	46	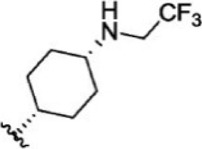	53	7.276
21	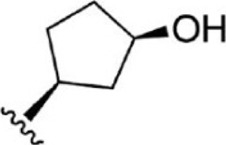	5.4	8.268	47	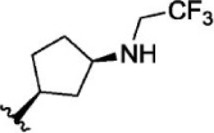	2	8.699
22	**-**	0.7	9.155	48	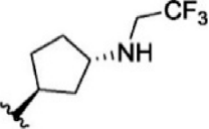	1.2	8.921
23	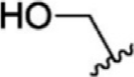	1.1	8.959	49	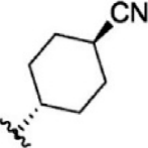	1.9	8.721
24	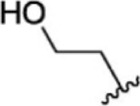	3.5	8.456	50	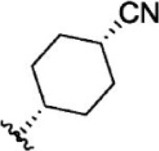	0.9	9.046
25	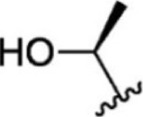	10	8.000	51	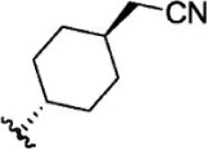	0.1	10.000
26	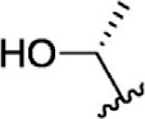	0.8	9.097	52	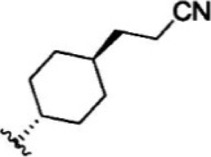	0.3	9.523

### Pharmacophore Model Generation

Phase 4.3, a high-performance program module of Schrödinger 2015, was used to generate the ligand-based pharmacophore models (Dixon et al., 2006). It uses a fine-grained conformational sampling method to predict a hypothesis consisting of the common pharmacophore features. The Ligprep module was used to clean, minimize, and generate conformations of all compounds. Based on the diversity of the chemical structure and its biological activity, the quantitative pharmacophore models were generated using the Develop Pharmacophore Model option. On the basis of biological activity distribution (pK_i_ values), the activity threshold value was set and the inhibitors were divided into actives, inactives, and moderately actives. In this study, both five and six featured pharmacophore hypotheses were generated by defining the minimum and maximum numbers of sites to five and six. The pharmacophore models were developed possessing different combinations of hydrogen-bond acceptor (A), hydrogen-bond donor (D), aromatic ring (R), hydrophobic group (H), positively ionizable (P), and negatively ionizable (N) groups. The resulting hypotheses were scored and ranked on the basis of scoring parameters. The scoring algorithm includes the alignment of site points and vectors, number of ligands matched, volume overlap, relative conformational energy, selectivity, and activity. The difference between the survival score and the survival inactive score notifies the ability of the hypotheses to correctly distinguish between actives and inactives.

### Pharmacophore Model Validation

Since the pharmacophore model is just a theoretical model, it is necessary to analyze whether or not the generated model is able to predict the active compounds. Thus, two approaches, namely, potency validation and selectivity validation, were performed to measure the accuracy of pharmacophores in selecting the active compounds.

#### Potency Validation

Potency validation was carried out to test whether the pharmacophore model is good enough to pick a greater number of active molecules. This was achieved by screening the database consisting of both active molecules and decoys. Active molecules are the known inhibitors of JAK with higher biological activities, whereas decoys are the molecule that does not have any activity toward JAK and it was downloaded from DUD-E (a Database of Useful Decoys-Enhanced) database (Mysinger et al., 2012). DUD-E datasets were used only after removing the biasness through docking. Based on the number of actives and decoys retrieved by the pharmacophore models, statistical parameters such as Guner-Henry (GH) score, %A, %Y, and E score were calculated using the following formula:

$$\text{GH score}=\;\left(\frac{Ha\;\left(3A\;+\;Ht\right)}{4*Ht*A}\right)\left(1-\;\frac{Ht\;-\;Ha}{D\;-\;A}\right);$$

$$\%A=\frac{Ha}{A}*100;\%Y=\frac{Ha}{Ht}*100;E=\frac{Ha/Ht}{A/D},$$

where Ha is the number of actives in the hits list, Ht is the number of hits retrieved, A is the number of active compounds in the database, D is the number of compounds in the database, %A is the percentage of known active compounds obtained from the database, %Y is the percentage of known actives in the hits list, and E is the enrichment of the concentration of actives by the model relative to random screening without a pharmacophoric approach. GH score ranges from 0 to 1, which indicates a null model and an ideal model, respectively. GH score >0.6 indicates the acceptable quality of the pharmacophore model and is useful in differentiating the known active molecules from inactives and suitable for retrieving active JAK1 inhibitors (Sathe et al., 2014; Li et al., 2015).

#### Selectivity Validation

Selectivity validation was performed to check which pharmacophore models are more selective in choosing high number of JAK1 molecules. Selectivity validation was carried out in two ways. First, a database comprising 30 JAK1, 30 JAK2, 30 JAK3, and 30 TYK2 molecules (Yang et al., 2007; Wang et al., 2009; Pissot-Soldermann et al., 2010; Ioannidis et al., 2011; Kulagowski et al., 2012) was created and used for validation. The ability of pharmacophore models to differentiate the selective JAK inhibitors was evaluated using virtual screening workflow on a manually curated database. Second, to further confirm the selectivity of the selected models, the available 288 JAK1 (Kulagowski et al., 2012; Labadie et al., 2012; Hurley et al., 2013; Labadie et al., 2013), 627 JAK2 (Lucet et al., 2006; Wang et al., 2009; Pissot-Soldermann et al., 2010; Harikrishnan et al., 2011; Ioannidis et al., 2011; Schenkel et al., 2011; Dugan et al., 2012; Forsyth et al., 2012; Lynch et al., 2013; Vazquez et al., 2018), and 431 JAK3 (Chrencik et al., 2010; Thoma et al., 2011; Jaime-Figueroa et al., 2013; Lynch et al., 2013; Soth et al., 2013; De Vicente et al., 2014; Duan et al., 2014) inhibitors from diverse research papers that mention either IC_50_ values or K_i_ values of these inhibitors were taken for validation.

### Pharmacophore-Based Virtual Screening

Virtual screening is the process where the complete databases are used to identify the molecules in the database which are most likely to bind to a drug target (Vyas et al., 2008). In this study, pharmacophore-based virtual screening was carried out using the “find matches to hypothesis” option available in the phase module which efficiently search for pharmacophore matches from the database of fixed conformers. The pharmacophore-based virtual screening was performed against Maybridge (53,000) (www.maybridge.com), Lifechemicals (12, 92, 000) (https://lifechemicals.com/), Enamine (24,91,318) (https://enamine.net/), Chemdiv (15,00,000) (https://www.chemdiv.com/), Asinex (398,022) (https://www.asinex.com/), and Zinc chemical and Zinc natural databases (https://zinc.docking.org/) (44,92,226) (Irwin and Shoichet 2005; Irwin et al., 2012; Sterling and Irwin 2015) to identify the new molecules with novel scaffolds. After screening, fitness score that is a measure of how well the hypothesis matched to the aligned ligand conformers based on RMSD site matching, volume terms, and vector alignments was used to filter the molecules.

### Absorption, Distribution, Metabolism, and Excretion Prediction

After virtual screening, the molecular descriptors and pharmaceutically applicable properties of the hits were calculated using Qikprop 4.4. Qikprop generates the physicochemical properties for a compound to find whether the compound follows drug likeliness properties. Lipinski’s rule characterizes the important molecular properties of drug, including absorption, distribution, metabolism, and excretion (ADME) that is essential for a drug’s pharmacokinetics in the human body (Jorgensen and Duffy 2002). Parameters that determine the ADME of the molecules were Molweight (Molecular weight), QPlogPo/w (partition coefficient), QPlogS (water solubility), percentage of human oral absorption, and intestinal absorption parameters such as Caco-2 and MDCK permeability. The compounds are expected to be active in humans only if the molecule passes through Lipinski’s rule of five. Therefore, the compounds retrieved after filtration were subjected to ADME prediction and its physicochemical properties were analyzed.

### Molecular Docking

Molecular docking predicts the binding mode and interaction of the small molecule to the protein. It distinguishes the behavior of small molecules in the binding site of target protein and explicates its fundamental biochemical processes (Gschwend et al., 1996; Lipinski 2000). The binding conformations of the hits inside the JAK1 ATP-binding site were investigated using Grid-based Ligand Docking with Energetics (Glide 6.7) module. The ATP-binding site of JAK1 comprises Leu881, Glu883, Val889, Ala906, Met956, Glu957, Phe958, Leu959, Gly962, Ser963, Glu966, Arg1007, Asn1008, Leu1010, Gly1020, and Asp1021 residues. Before docking, protein preparation wizard was used to prepare protein structure (3EYG) (Williams et al., 2009) applying the default parameters that include adding hydrogens, filling missing atoms and residues using PRIME, assigning correct bond orders, and hydrogen-bond optimization and minimization. In the Receptor Grid Generation panel, the center of the gird box was defined on the centroid of the co-crystallized ligand (MI1), and the volume in the active-site region of the receptor was calculated by default settings (van der Waals radius scaling factor 1.0 and partial charge cutoff 0.25). Molecular docking was performed using both the Standard Precision (SP) and Extra Precision (XP) docking modes in which the receptor was held rigid and the ligand was free to move (Jain 2003; Halgren et al., 2004). Glide score is a combination of hydrophobic, hydrophilic, van der Waals energy, metal binding groups, freezing rotatable bonds, and polar interactions with the receptor. Comparing Glide SP and XP score, Glide SP score is a softer and more forgiving function whereas Glide XP score is a harder function and adept at reducing the false positives. Therefore, Glide XP score was considered for the selection of hits and further analysis.

### MM-GBSA Calculations

The binding free-energy calculations procured via the MM-GBSA method are more precise and consistent than the glide XP score and improve the ranking of potential leads (Lyne et al., 2006; Das et al., 2009; Yang et al., 2009). Therefore, the binding free energy (∆G bind) of the protein–ligand complexes was calculated using the Prime MM-GBSA module implemented in Schrödinger 2015. The Prime MM-GBSA Module incorporates the OPLS3 force field and the VSGB dissolvable model to look through calculations (Li et al., 2011). The energy difference between the free and complex states of protein and ligand was calculated. The energy components such as covalent binding energy, van der Waals energy, generalized born electrostatic solvation energy, Coulomb energy, total energy, and H-bond correction were retrieved from the calculations.

### Induced Fit Docking

In the docking protocol, to retain the flexibility of the receptor, a mixed molecular docking protocol called induced fit docking (IFD) developed by Schrödinger 2015 was employed (Wang H. Y. et al., 2008). IFD uses the refinement module in Prime to account for the receptor flexibility and Glide to account for the ligand flexibility (Jacobson et al., 2004). Protein preparation wizard and the Ligprep module were used for protein and ligand preparation, respectively. Grid was generated on the ATP-binding site amino acid residues based on the co-crystallized ligand. The ATP-binding site residues and their flexibility were considered for the IFD protocol. IFD was carried out with default parameters, and 20 conformational poses were calculated for each ligand. IFD scores (IFD score = 1.0 Glide_Gscore +0.05 Prime_Energy) were calculated based upon the total energy of the system and the protein–ligand interaction energy and used to rank the IFD poses (Luo et al., 2014). The electrostatic interactions formed between the receptor and the ligand were calculated by the docking scores under “Electro,” and hydrophilic interactions under “Lipophilic Evdw” mention the lipophilicity component acquired from the hydrophobic grid. IFD poses were ranked based on the scores, and the best pose was chosen for each hit.

### Cross Docking

Cross docking is the process of taking a series of complexes of ligand–receptor pairs and docking every ligand to every receptor. This is used to study the specificity of the ligands and the receptors and, thus, yield valuable report regarding the effects of ligand upon binding. Protein preparation wizard and LigPrep were used to prepare proteins and the shortlisted hits, respectively. Grid was generated on the ATP-binding site residues. The hits shortlisted from the molecular docking study were docked against JAK1, JAK2, and JAK3 using the Glide XP module to identify the selective lead compounds.

### Molecular Dynamics Simulation

Docking results could be the instantaneous state and were not considered decisive because binding of the inhibitor to a protein in an *in vivo* state is a dynamic process. For advanced studies, the stable binding mode of the ligand is more reliable. Hence, to explore the detailed binding modes and compare the stability and molecular interactions of the docked lead complexes, molecular dynamics simulation was carried out for 100ns using GROningen MAchine for Chemical Simulations (GROMACS version 2016.3 installed in Centos 7.3) software (Abraham et al., 2015). GROMACS works according to Newton’s laws of motions and simulates the behavior of bio-molecules such as nucleic acids, proteins, lipids, ligands, ions, and water. The coordinates for MD simulations have been achieved from the docking results. The PRODRG server (http://davapc1.bioch.dundee.ac.uk/cgi-bin/prodrg) was used to calculate the ligand parameters in the framework of GROMOS96 54a7 force field. The SPC water model was used as a solvent during simulation. To achieve the stability of the simulated system, the potential energy, temperature, and pressure were monitored during the simulations. The temperature and pressure of the system were equilibrated (from ps to ns) till they reach 300 K and 1.05 bar, respectively. The stability of the secondary structure elements and conformational changes of the simulated complexes were evaluated by root mean square deviations (RMSDs), root mean square fluctuation (RMSF), radius of gyration (Rg), and solvent-accessible surface area (SASA) values obtained from MD trajectories. The molecular dynamics study was performed using High-Performance Computing server (Intel Xeon 14 core processor with 28 threads and 2.40 GHz processor speed).

### MM-PBSA Calculation

The molecular mechanics energies combined with the Poisson–Boltzmann and surface area continuum solvation (MM/PBSA) method have been applied to predict binding free energies and to evaluate the relative stabilities of different bimolecular structures. The MM/PBSA calculations were performed for the simulated systems using g_mmpbsa, a GROMACS Tool for High-Throughput MM-PBSA Calculations (Kumari et al., 2014). Combined with molecular dynamics (MD) simulations, MM-PBSA can also incorporate conformational fluctuations and entropic contributions to the binding energy (Homeyer and Gohlke 2012).

### Density Functional Theory Study

The density functional theory (DFT) study was carried out to observe the chemical behavior of the lead compounds using the electron density-relevant concepts (Zhao et al., 2011). Also, it provides a quantum-level understanding of the molecules and assists in building the relationship between the electronic properties and the biological activity of the molecule (Nagarajan et al., 2018). Molecular descriptors such as total energy, highest occupied molecular orbital (HOMO), lowest unoccupied molecular orbital (LUMO), band energy gap (ΔE), molecular dipole moment, absolute hardness (η), global softness (σ), chemical potential (μ), electronegativity (χ), and electrophilicity index (ω) were studied for the selected lead compounds using Gaussian 16 software. Initially, the molecules were optimized using the B3LYP function with a 6-31G(d) basis set to calculate their molecular properties such as total energy and molecule dipole moment (Becke 1998). The dipole moment relates to the electro-chemical reactivity of the compounds. The electron donating and accepting ability of the molecules HOMO energy (E_HOMO_) and LUMO energy (E_LUMO_), respectively, were calculated.

## Results and Discussion

### Pharmacophore Model Generation

In the phase module, ligand-based pharmacophore model generation was carried out utilizing 52 JAK1 inhibitors named C-2 methyl/hydroxyethyl imidazopyrrolopyridine derivatives (Table 1) along with their activity values. Ten molecules whose pK_i_ > 8.9 were taken as actives, twelve molecules whose pK_i_ < 8.1 were taken as inactives, and the remaining thirty-one molecules were considered to be intermediates. Twenty-seven different pharmacophore hypotheses (six with six featured pharmacophores and twenty-one with five featured pharmacophores) were generated and put through the stringent scoring function. The generated pharmacophores were ranked by aligning them with the active ligands, and the statistical data obtained after scoring are tabulated in Table 2. Besides the survival active score, survival inactive score, and post-hoc score, fitness score was considered to measure the quality of the pharmacophores. The fitness score was calculated between the pharmacophores and the highly active (compound 51) and highly inactive (compound 7) compounds in the dataset. For all pharmacophores, the fitness score was higher with the highly active compound compared with the inactive compound. Subsequently, the pharmacophores were evaluated using different validation methods.

**TABLE 2:** The summary of statistical data obtained for the pharmacophore hypotheses.

S. no.	Hypothesis	Survival score	Survival inactive	Post hoc	Site	Vector	Volume
1	**ADHRRR**	**3.514**	**1.420**	**3.514**	**0.72**	**0.992**	**0.803**
2	AADHRR	3.513	1.396	3.513	0.72	0.994	0.801
3	AAADHR	3.509	1.410	3.509	0.71	0.995	0.805
4	**DDHRRR**	**3.424**	**1.371**	**3.424**	**0.77**	**0.953**	**0.705**
5	ADDHRR	3.420	1.364	3.420	0.75	0.960	0.710
6	AADDHR	3.398	1.244	3.398	0.73	0.948	0.724
7	AADRR	4.378	2.106	3.607	0.85	0.992	0.761
8	AAADR	4.364	2.141	3.593	0.84	0.969	0.780
9	**ADRRR**	**4.353**	**2.081**	**3.582**	**0.85**	**0.979**	**0.757**
10	ADDRR	4.339	2.080	3.568	0.89	0.917	0.763
11	AADDR	4.330	1.877	3.559	0.88	0.920	0.755
12	**DDRRR**	**4.292**	**1.841**	**3.521**	**0.87**	**0.901**	**0.747**
13	ADHRR	4.259	1.747	3.487	0.80	0.979	0.709
14	**DHRRR**	**4.257**	**1.883**	**3.486**	**0.81**	**0.969**	**0.710**
15	AADHR	4.253	1.744	3.481	0.79	0.985	0.709
16	AHRRR	3.974	1.916	3.510	0.72	0.988	0.803
17	AAHRR	3.972	1.892	3.508	0.72	0.991	0.801
18	AAAHR	3.965	1.907	3.501	0.70	0.993	0.806
19	AAADH	3.954	1.927	3.490	0.69	0.996	0.802
20	**DPRRR**	**3.945**	**1.666**	**3.481**	**0.71**	**0.993**	**0.780**
21	**ADPRR**	**3.940**	**1.662**	**3.476**	**0.71**	**0.993**	**0.776**
22	AADPR	3.929	1.631	3.465	0.69	0.993	0.778
23	ADDHR	3.896	1.788	3.432	0.73	0.959	0.741
24	**DDHRR**	**3.896**	**1.794**	**3.432**	**0.78**	**0.951**	**0.701**
25	AADDH	3.853	1.726	3.389	0.73	0.957	0.701
26	DHHRR	3.441	1.703	2.977	0.44	0.964	0.575
27	ADHHR	3.437	1.612	2.973	0.46	0.961	0.555

**A**- acceptor, **D**- donor, **H**- hydrophobic, **R**- aromatic ring, and **P**- positive group. The selected pharmacophore hypotheses are represented in bold.

### Pharmacophore Model Validation

#### Potency Validation

For potency validation, a database containing 40 JAK1 actives and 1,000 decoys was created. The generated pharmacophore models were allowed to screen this database to calculate the GH score. It was observed that six featured hypotheses have picked less number of decoys compared with five featured hypotheses. The ADHRRR, DDHRRR, and DPRRR hypotheses were more potent because they do not pick any decoys. DDRRR, ADPRR, and AHRRR have picked very less number of decoys. DDRRR, ADHRR, DHRRR, AADHR, DDHRR, and DDRRR have picked more active molecules. The results of potency validation are tabulated in Table 3. Based on the number of actives and decoys retrieved by the hypotheses, the GH score was calculated. The hypotheses such as DDHRRR, ADHRRR, ADDHRR, DDRRR, DPRRR, DHRRR, ADRRR, AHRRR, DDHRR, and ADPRR have obtained the GH score >0.6 indicating the goodness of these hypotheses.

**TABLE 3:** Pharmacophore validation results from potency validation.

S. no.	Hypothesis	H_a_ (#40)	Decoys (#1000)	H_t_	%A	%Y	E	GH score
1	**ADHRRR**	**11**	**0**	**11**	**27.50**	**100.00**	**26.00**	**0.819**
2	AADHRR	11	10	21	27.50	52.38	13.62	0.457
3	AAADHR	11	22	33	27.50	33.33	8.67	0.312
4	**DDHRRR**	**21**	**0**	**21**	**52.50**	**100.00**	**26.00**	**0.881**
5	ADDHRR	24	13	37	60.00	64.86	16.86	0.628
6	AADDHR	22	27	49	55.00	44.90	11.67	0.461
7	AADRR	23	106	129	57.50	17.83	4.64	0.248
8	AAADR	21	242	263	52.50	7.98	2.08	0.145
9	**ADRRR**	**23**	**9**	**32**	**57.50**	**71.88**	**18.69**	**0.677**
10	ADDRR	26	39	65	65.00	40.00	10.40	0.444
11	AADDR	22	128	150	55.00	14.67	3.81	0.216
12	**DDRRR**	**28**	**1**	**29**	**70.00**	**96.55**	**25.10**	**0.898**
13	ADHRR	33	85	118	82.50	27.97	7.27	0.381
14	**DHRRR**	**31**	**12**	**43**	**77.50**	**72.09**	**18.74**	**0.726**
15	AADHR	32	252	284	80.00	11.27	2.93	0.213
16	AHRRR	11	3	14	27.50	78.57	20.43	0.656
17	AAHRR	11	78	89	27.50	12.36	3.21	0.149
18	AAAHR	11	141	152	27.50	7.24	1.88	0.106
19	AAADH	11	54	65	27.50	16.92	4.40	0.185
20	**DPRRR**	**11**	**0**	**11**	**27.50**	**100.00**	**26.00**	**0.819**
21	**ADPRR**	**11**	**4**	**15**	**27.50**	**73.33**	**19.07**	**0.616**
22	AADPR	12	10	22	30.00	54.55	14.18	0.479
23	ADDHR	23	71	94	57.50	24.47	6.36	0.304
24	**DDHRR**	**29**	**17**	**46**	**72.50**	**63.04**	**16.39**	**0.643**
25	AADDH	19	132	151	47.50	12.58	3.27	0.185
26	DHHRR	23	53	76	57.50	30.26	7.87	0.351
27	ADHHR	19	164	183	47.50	10.38	2.70	0.164

The number of compounds used for the validation study is mentioned within parenthesis. The selected pharmacophore hypotheses are represented in bold.

#### Selectivity Validation

Initially, the selectivity validation was performed with a set of 30 JAK1, 30 JAK2, 30 JAK3, and 30 TYK2 molecules retrieved from different studies. DPRRR has picked only JAK1 molecules. The DHRRR, ADHRR, DDRRR, and DDHRR hypotheses have picked a high number of JAK1 molecules and very less JAK3 molecules. ADHRRR and DDHRRR have picked only few JAK1 and one JAK3 molecules. The results of selectivity validation are tabulated in Table 4. The fitness score for JAK1 inhibitors was greater than or equal to 1.5, whereas for other JAK inhibitors, the fitness score was <1.5 for most of the molecules indicating that the pharmacophore models were able to map well with the JAK1 inhibitors (Sathe et al., 2014; Babu et al., 2015).

**TABLE 4:** Pharmacophore validation results from selectivity validation.

S. no.	Hypothesis	No. of inhibitors retrieved						
		JAK1 (#30)	JAK2 (#30)	JAK3 (#30)	TYK2 (#30)	JAK1 (#288)	JAK2 (#627)	JAK3 (#431)
1	**ADHRRR**	**5**	**-**	**1**	**-**	**24**	**0**	**1**
2	AADHRR	**-**	6	1	**-**	**-**	**-**	**-**
3	AAADHR	**-**	4	**-**	**-**	**-**	**-**	**-**
4	**DDHRRR**	**8**	**-**	**1**	**-**	**54**	**0**	**1**
5	ADDHRR	1	9	1	**-**	**-**	**-**	**-**
6	AADDHR	2	9	**-**	**-**	**-**	**-**	**-**
7	AADRR	7	16	3	3	**-**	**-**	**-**
8	AAADR	4	9	1	**-**	**-**	**-**	**-**
9	**ADRRR**	**10**	**-**	**3**	-	**77**	**22**	**5**
10	ADDRR	3	**-**	4	**-**	**-**	**-**	**-**
11	AADDR	2	9	1	**-**	**-**	**-**	**-**
12	**DDRRR**	**18**	**-**	**2**	-	**86**	**1**	**10**
13	ADHRR	23	**-**	3	**-**	**-**	**-**	**-**
14	**DHRRR**	**26**	**-**	**1**	-	**142**	**56**	**20**
15	AADHR	21	13	1	3	**-**	**-**	**-**
16	AHRRR	**-**	**-**	7	**-**	**-**	**-**	**-**
17	AAHRR	**-**	13	1	**-**	**-**	**-**	**-**
18	AAAHR	**-**	8	1	**-**	**-**	**-**	**-**
19	AAADH	**-**	4	**-**	**-**	**-**	**-**	**-**
20	**DPRRR**	**9**	**-**	**-**	-	**19**	**0**	**0**
21	**ADPRR**	**9**	**2**	**-**	-	**25**	**0**	**0**
22	AADPR	9	1	**-**	**-**	**-**	**-**	**-**
23	ADDHR	4	9	**-**	**-**	**-**	**-**	**-**
24	**DDHRR**	**17**	**-**	**1**	b	**60**	**6**	**37**
25	AADDH	**-**	9	**-**	**-**	**-**	**-**	**-**
26	DHHRR	17	13	3	**-**	**-**	**-**	**-**
27	ADHHR	12	23	4	3	**-**	**-**	**-**

The number of compounds used for the validation study is mentioned within parenthesis. The selected pharmacophore hypotheses are represented in bold.

**FIGURE 1:**
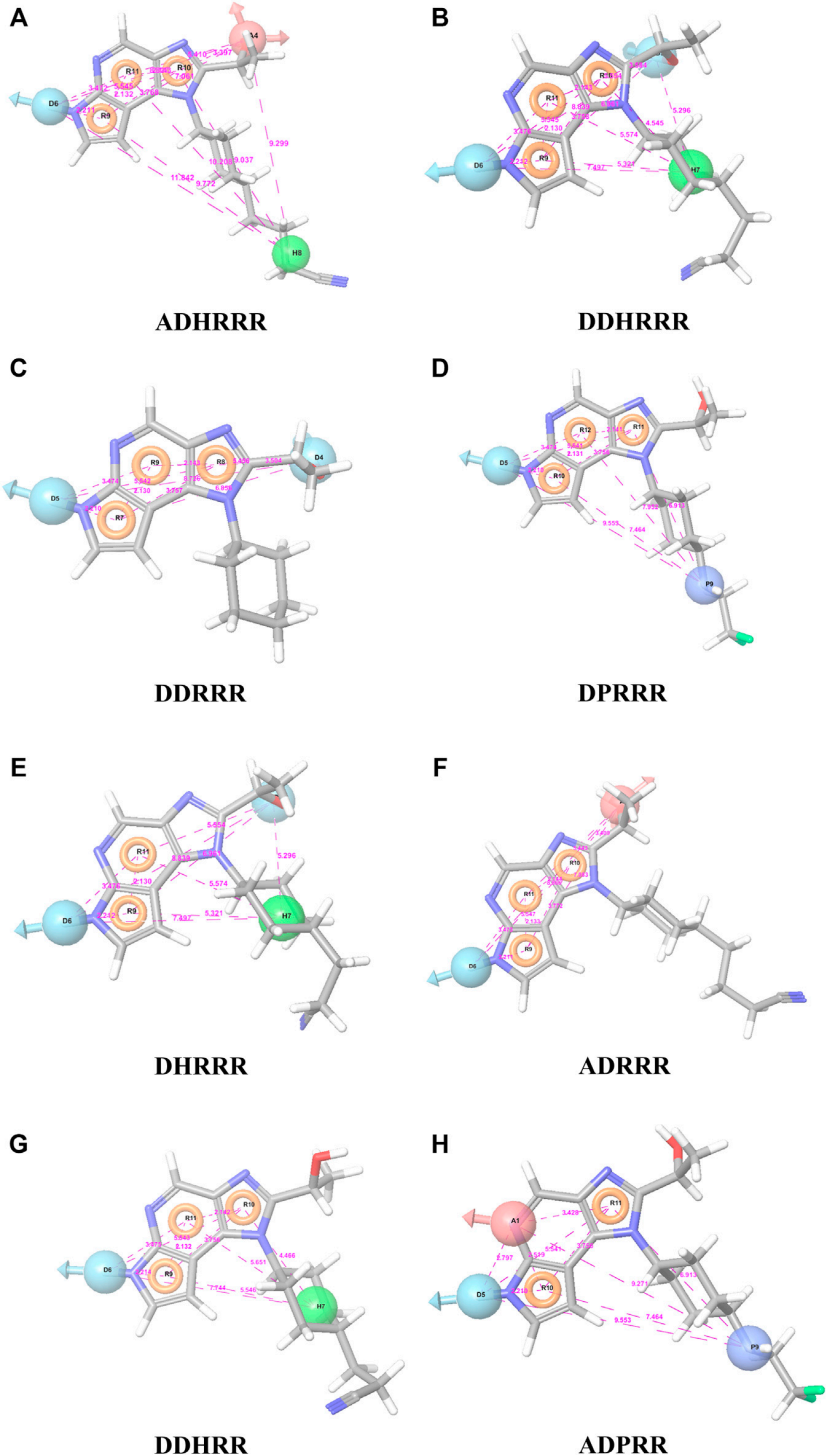
The representation of selected pharmacophore models **(A)** ADHRRR, **(B)** DDHRRR, **(C)** DDRRR, **(D)** DPRRR, **(E)** DHRRR, **(F)** ADRRR, **(G)** DDHRR, and **(H)** ADPRR. Pharmacophore features are colored in light blue, brown, dark blue, brick red, and green contours representing the H-bond donor (D), H-bond acceptor (A), positives (P), aromatic ring (R), and hydrophobic (H) groups, respectively. The distances between the pharmacophore features (A˚) are given in pink dotted lines.

Six feature pharmacophore hypotheses were more potent but not highly selective to JAK1. Based on potency and selectivity validation results, the DDHRRR, ADHRRR, DDRRR, DPRRR, DHRRR, ADRRR, DDHRR, and ADPRR hypotheses were selected because they were successful in retrieving active compounds from the database. The representation of the selected JAK1 pharmacophore models showing the distances between the pharmacophoric sites is shown in Figures 1A–H. On mapping the selected pharmacophore models with highly active compound 51 and inactive compound 7, it was observed that the fitness score was >2.5 for the highly active compound mapping with all pharmacophore features whereas inactive compound 7 could map with either four or five pharmacophore features with low fitness score. The highest fitness score with compound 51 suggests screening using these models would pick the similar active compounds. From the results, we suggest the combination of two or three aromatic rings (R) and one or two donor atoms (D) with a hydrophobic (H) group is an important pharmacophoric feature for identifying the selective JAK1 inhibitors. The important pharmacophore features obtained were compared with the contribution maps obtained through the hologram-based fingerprint technique (Supplementary Figure S1). The contribution maps depict the imidazopyrrolopyridine ring which possesses one donor and three aromatic rings responsible for the intermediate contribution of the inhibitory activity. From the highly active compounds, we observed the cyano group attached to cyclohexanes (yellow) and the hydroxyethyl group attached to imidazopyrrolopyridines (green); a hydrophobic and an aromatic/donor group, respectively, are strongly responsible for the higher activity. Thus, these pharmacophore features are highly important for the inhibitory activity of JAK1.

To confirm the selectivity of the selected pharmacophore models (DDHRRR, ADHRRR, DDRRR, DPRRR, DHRRR, ADRRR, DDHRR, and ADPRR), the second round of selectivity validation was carried out with a set of 288 JAK1, 627 JAK2, and 431 JAK3 inhibitors with diverse activity. It was observed (Table 4) that all the selected pharmacophore models were able to pick more number of JAK1 inhibitors compared with its subtypes. Hence, the selected pharmacophore models were capable of discriminating the JAK1 inhibitors and appropriate for retrieving the novel and selective JAK1 inhibitors.

### Pharmacophore-Based Virtual Screening

The selected pharmacophore models were screened against Maybridge, Lifechemicals, Enamine, Chemdiv, Asinex, and Zinc (chemical and natural) databases for the identification of new hits. The identified hits contain the structural features that overlap with the selected pharmacophore models. The hits obtained were ranked and filtered based on the fitness score. The fitness score was set to >1.5 for the Maybridge, Asinex, Chemdiv, Lifechemicals, and Enamine databases, whereas for the Zinc database, the fitness score was set to >2 because of high number of molecules retrieved from the Zinc database. As a result of screening and filtration, 4,265 compounds were retrieved. The total numbers of hits retrieved from different databases are tabulated in Table 5.

**TABLE 5:** Number of hits obtained from pharmacophore-based virtual screening.

S. no.	Hypothesis	Maybridge	Lifechemicals	Enamine	Asinex	Chemdiv	Zinc
1	ADHRRR	0	10	5	16	4	0
2	DDHRRR	0	6	9	0	1	0
3	DDRRR	3	117	167	18	16	16
4	DPRRR	1	57	68	9	40	14
5	DHRRR	9	250	321	161	151	155
6	ADRRR	3	134	224	335	17	113
7	DDHRR	9	151	558	44	88	213
8	ADPRR	1	74	436	30	9	202

The potentiality of the pharmacophore models was validated using receiver operating curves (ROCs) utilizing the screened molecules (Hevener et al., 2009); 10 compounds identified from the pharmacophore-based virtual screening were seeded with 500 decoys. Enrichment was estimated based on how well the compounds were fetched. After ranking the decoy set and docked compounds by the Glide score, the enrichment was calculated using the ROC plot that provides the report on sensitivity and specificity. The ROC plot inferred that Glide XP ranked seven compounds in top 10% with the ROC value as 0.93 and the AUC value as 0.92. 80% of the true positives were fetched in top 20% which indicates its capability of retrieving the active compounds. The gentle increase in the ROC curve (Supplementary Figure S2) was noticed in the beginning, which implies that number of true positives was sacrificed to reduce the amount of false positives.

### Absorption, Distribution, Metabolism, and Excretion Prediction

Compounds that pass Lipinski’s rule of five and other ADME properties of the drug are expected to be active in humans. Properties such as molecular weight, H-bond donors, H-bond acceptors, log p, van der Waals surface, aqueous solubility, blockage of HERG K+ channels, apparent Caco-2 cell permeability, apparent MDCK cell permeability, brain/blood partition coefficient, skin permeability, binding to human serum albumin, and human oral absorption of the hits were studied. Finally, 2,856 compounds whose drug-like properties were in the acceptable range (according to qikprop recommended range) were selected and subsequently exposed to glide SP and XP docking protocols to remove both the false-positive and false-negative hits.

### Molecular Docking

**FIGURE 2:**
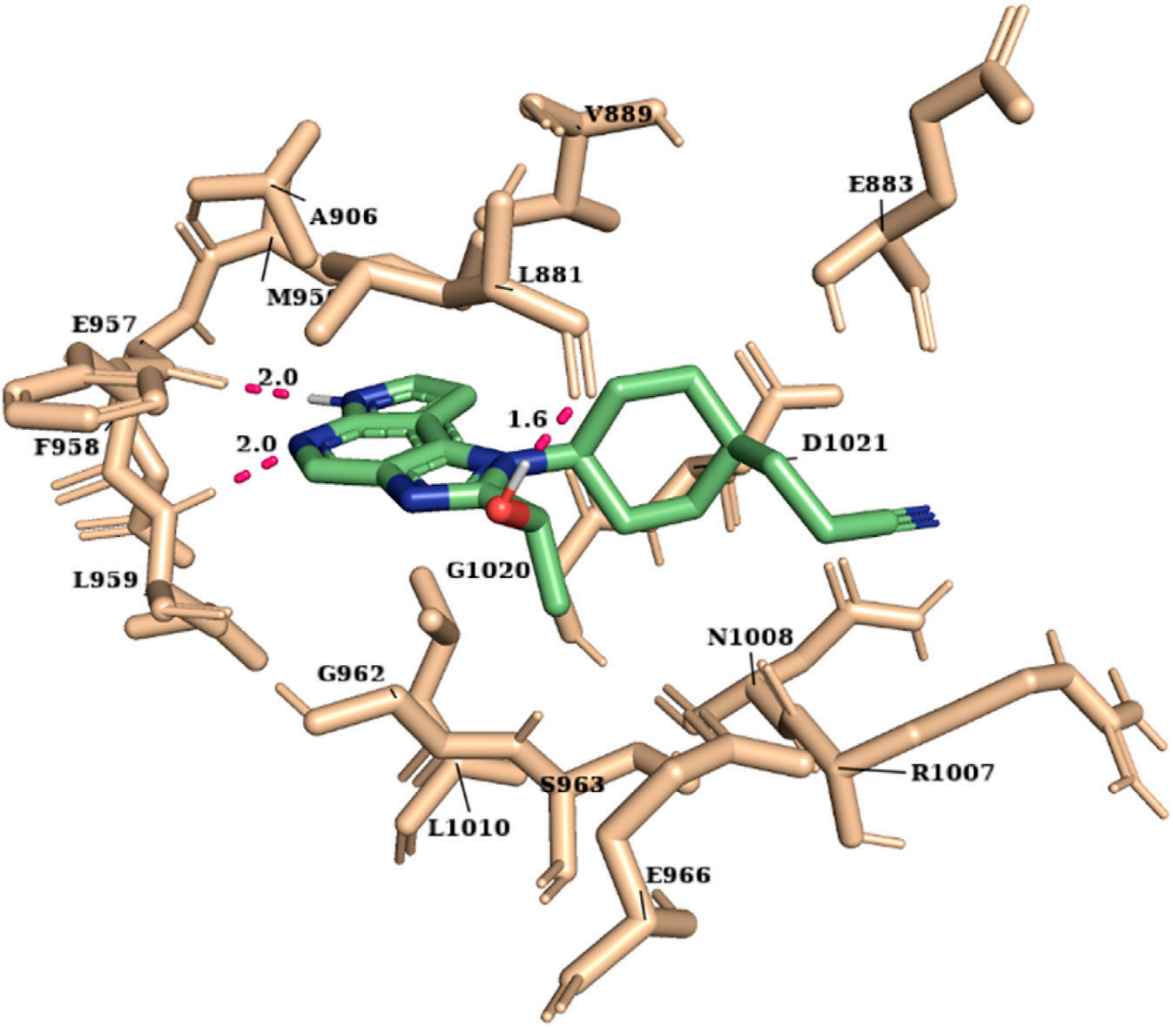
The binding of highly active compound 51 into the ATP-binding site of JAK1.

The molecular docking study was carried out using the Glide SP and XP modes to explore the binding mode and interaction of hits on the ATP-binding site. The crystal structure of JAK1 protein 3EYG in complex with MI1 (Williams et al., 2009) was used to perform molecular docking. The grid was developed on the centroid of co-crystallized ligand MI1 surrounding the ATP-binding site residues (Leu881, Glu883, Val889, Ala906, Met956, Glu957, Phe958, Leu959, Gly962, Ser963, Glu966, Arg1007, Asn1008, Leu1010, Gly1020, and Asp1021) of JAK1. Initially, the docking of MI1 into the ATP-binding site was performed to check the accuracy and reproducibility of the docking program. Subsequently, the highly active compound 51 and 2,856 hits were docked into the ATP-binding site. Considering the docking result of compound 51 (glide XP score -9.691), the glide XP threshold value was set to ≥ −9.60 to identify the novel hits. We observed that 90 molecules have exhibited glide score greater than the threshold and it was shortlisted (Supplementary Table S1). Among the JAK1 ATP-binding site residues, Leu959 and Glu957 that are present in the hinge region were found to be the most selective amino acid residues for the H-bond interaction and also crucial for selective inhibition of JAK1. Hence, the interactions with Leu959 and Glu957 were investigated for the hits. Compound 51 has shown H-bond interactions with Leu959, Glu957, and Leu881. The selected 90 hits have exhibited H-bond interaction with either Leu959 or Glu957 or both residues. Additionally, the Leu881, Glu883, Ser963, Glu966, and Arg1007 residues were involved in H-bond interaction with most of the hits. The hydrophobic interactions were formed mainly by the residues Leu881, Val889, Ala906, Val938, Met956, Phe958, Pro960, and Leu1010. The binding of compound 51 into the ATP-binding site is shown in Figure 2.

### MM-GBSA Calculations

The highly ranked hits selected from glide docking were taken for MM-GBSA calculations to predict the binding energy of the protein–ligand complexes. The calculated free energy of binding (∆G bind) was lower than glide energy. It was observed that van der Waals (∆G_Bind_vdW) energy contributes more for the ligand binding, whereas covalent interaction (∆G_Bind_Covalent) and electrostatic salvation (∆G_Bind_Solv_GB) energy terms disfavor for the inhibitor binding.

### Induced Fit Docking

IFD was performed on the highly ranked hits using the crystal structure of JAK1 (3EYG). It was observed that IFD also produces good IFD score and XP score comparable to glide XP score. The IFD score of JAK1 hits was greater than or equal to −590, and their corresponding XP score was greater than −8.00 which indicates the good binding ability of the hits. The observed H-bond and hydrophobic interaction with the IFD results was highly similar to glide results, indicating that these hits could bind and produce similar H-bond and hydrophobic interaction inside the binding site upon both receptor and ligand flexibility.

### Cross Docking

Since an important objective of this work is to attain admissible levels of intra-family selectivity, the cross-docking approach was employed for the highly ranked hits. For cross docking, the crystal structure of JAK1 (3EYG), JAK2 (3KRR), and JAK3 (3ZEP) was used. Among 90 hits tested, the top five compounds (T6649932, ST088474, T5923555, T5923531, and T6763842) that have the highest docking score toward JAK1 (>-10.5) compared with JAK2 and JAK3 were selected and taken for further study.

### Analysis of Selected Lead Compounds

**FIGURE 3:**
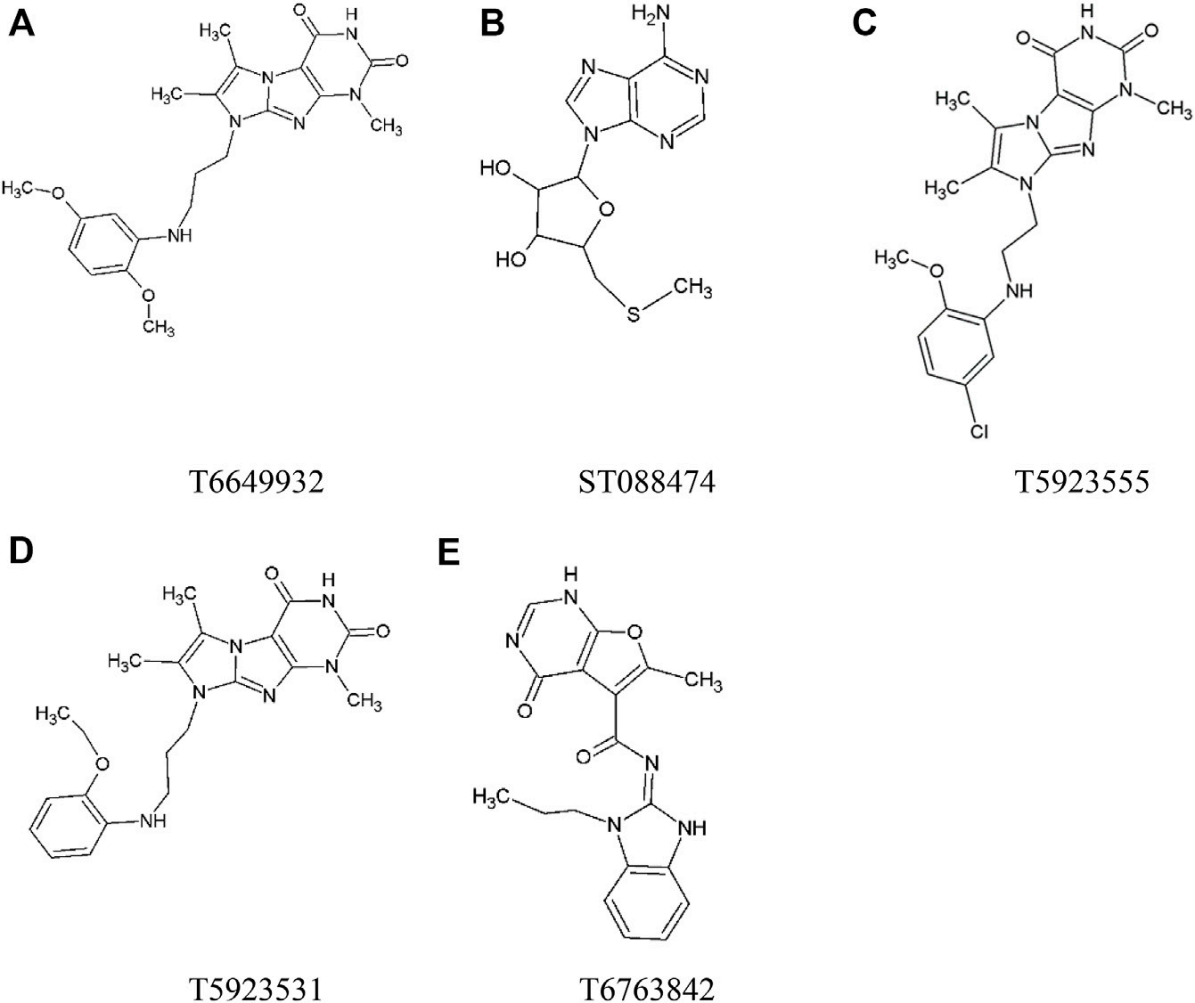
The chemical structure of selected lead compounds. **(A)** T6649932, **(B)** ST088474, **(C)** T5923555, **(D)** T5923531, and **(E)** T6763842.

The top five compounds that showed good potency and selectivity were selected and analyzed. To identify the potency and selectivity of the leads, a drug molecule named ruxolitinib was included in the study. Molecular docking, MM-GBSA calculations, IFD docking, and cross docking were performed for the drug and compared with the selected leads. Subsequently, the selected leads were taken for the molecular dynamics simulation study using the GROMACS and DFT calculations using Gaussian. The chemical structures of the selected lead compounds are shown in Figures 3A–E.

#### Absorption, Distribution, Metabolism, and Excretion Properties

ADME properties are the key determinants for the successful development of new drugs. All the analyzed pharmacokinetic parameters of these lead compounds were found to be within the permissible range. The percentage of the human oral absorption was found to be greater than 50%. The partition coefficient and water solubility that are important for the assessment of absorption and distribution of drugs within the body ranged between −0.4 and 3.5 and −5.4 and −2.1, respectively. Compounds T6649932, T5923555, and T5923531 possessing good Caco-2 and MDCK permeability have good level of intestinal absorption. The drug-likeliness properties of the selected leads and ruxolitinib are given in Table 6.

**TABLE 6:** The drug likeliness properties of the selected lead compounds and the drug.

S. no.	Molecule ID	molMW (130.0–725.0)	dHB (0.0–6.0)	aHB (2.0–20.0)	logPo/w (–2.0–6.5)	logS (–6.5–0.5)	logBB (–3.0–1.2)	PCaco (<25 poor>500 great)	PMDCK (<25 poor>500 great)
1	T6649932	426.5	2	8	3.5	−5.3	−1.1	756.168	365.722
2	ST088474	297.3	4	10	−0.4	−2.1	−1.3	132.825	95.256
3	T5923555	416.9	2	7	3.3	−5.1	−0.8	582.955	674.629
4	T5923531	410.5	2	7	3.4	−5.4	−1.1	679.576	325.851
5	T6763842	351.4	1	6	2.7	−4.4	−1.2	385.229	176.425
6	Ruxolitinib	306.4	2	4.5	1.4	−3.3	−0.4	941.735	463.628

molMW, molecular weight; dHB, donor atoms; aHB, acceptor atoms; logPo/w, partition coefficient; logS, aqueous solubility; logBB, brain/blood partition coefficient; PCaco, predicted apparent; Caco-2, cell permeability in nm/sec; PMDCK, predicted apparent MDCK cell permeability in nm/sec. The qikprop recommended values are given inside the parenthesis.

#### Glide XP Docking Analysis

**FIGURE 4:**
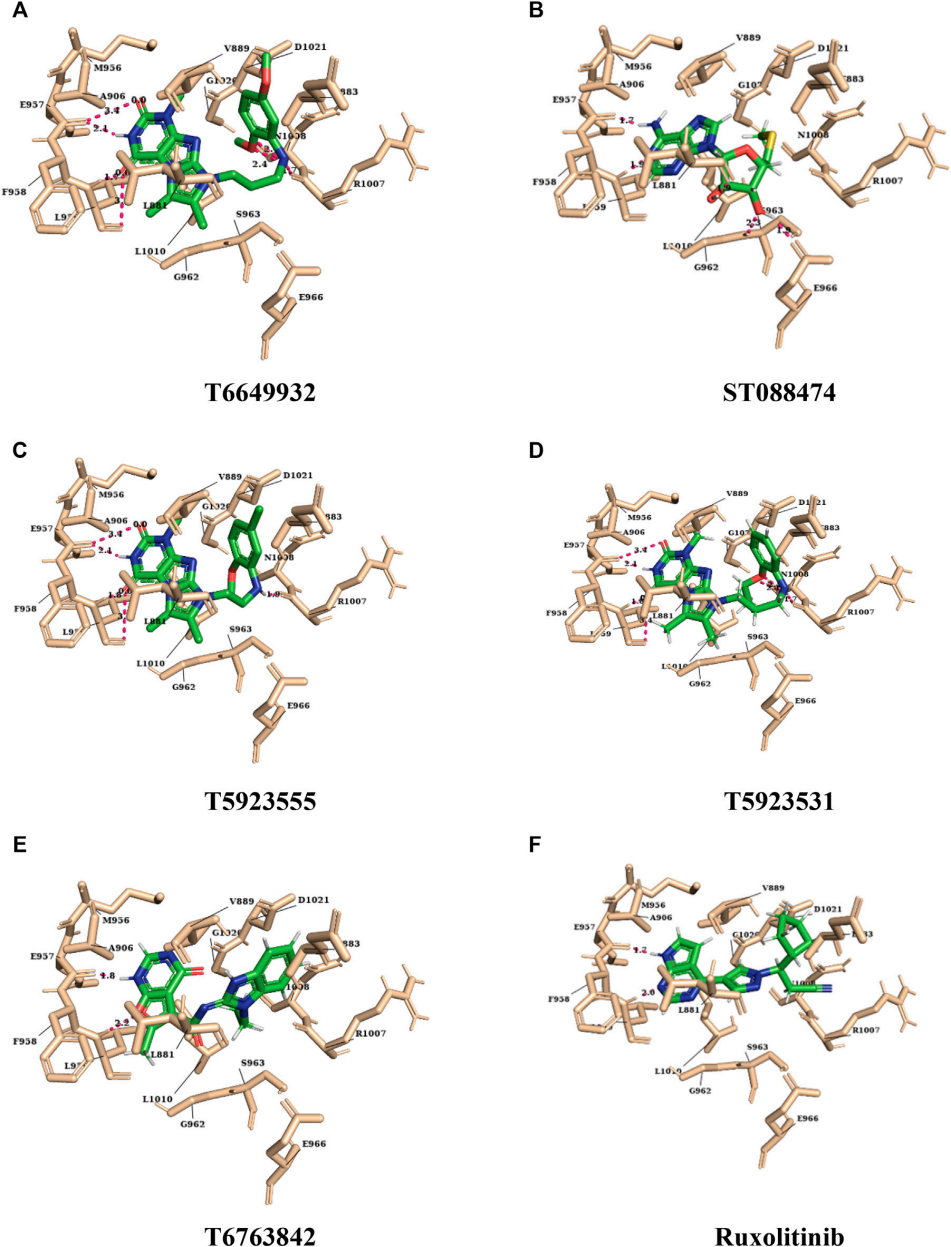
The representation of docked lead compounds and drug (**(A)** T6649932, **(B)** ST088474, **(C)** T5923555, **(D)** T5923531, **(E)** T6763842, and **(F)** ruxolitinib) present inside the ATP-binding site of JAK1 after molecular docking.

For the selected leads, the glide XP score was greater than −10.015, whereas for ruxolitinib, it was −9.282. The highest docking score was observed for T6763842 (−10.671). The major contribution of vdW interactions was observed which indicate that the vdW interaction favors the protein-ligand complex. The glide XP score, glide energy, glide evdw, and glide ecoul of the selected leads are given in Table 7. On analyzing the interaction, it was observed that the lead compounds showed conserved H-bond interactions with both the selective residues of JAK1 (Leu959 and Glu957) similar to the drug indicating its remarkable selectivity. Compounds such as T6649932, T5923555, and T5923531 formed another H-bond with Arg1007. Additionally, hydrophobic interactions were formed with the ATP-binding site residues Leu881, Val889, Ala906, Val938, Met956, Phe958, Pro960, and Leu1010. Figures 4A–F show the docked pose of lead compounds and drug inside JAK1 ATP-binding site.

**TABLE 7:** Molecular docking results of the selected JAK1 lead compounds and the drug.

S. no.	Molecule ID	XP score	Glide energy	Glide evdw	Glide ecoul	H-bond interaction
1	T6649932	−10.335	−61.771	−53.839	−7.932	Leu959, Glu957, Arg1007
2	ST088474	−10.653	−50.800	−34.600	−16.200	Leu959, Glu957, Leu881, Ser963, Glu966
3	T5923555	−10.015	−57.500	−49.348	−8.151	Leu959, Glu957, Arg1007
4	T5923531	−10.303	−57.350	−49.885	−7.465	Leu959, Glu957, Arg1007
5	T6763842	−10.671	−51.703	−46.166	−5.536	Leu959, Glu957
6	Ruxolitinib	−9.282	−57.553	−43.488	−14.065	Leu959, Glu957

#### MM-GBSA Analysis

The binding free energy of the selected lead compounds ranges from −41.698 to −46.430 suggesting good binding affinity with JAK1. Many lead compounds have shown comparable free energy of binding with ruxolitinib. This provides the insight that these lead compounds have exhibited good specificity. Furthermore, the contribution of ∆G_Bind_vdW and ∆G_Bind Lipo components to the binding free energy was compared. The high binding free energy was majorly contributed by ∆G_Bind vdW than ∆G_Bind Lipo component. The predicted binding free energy of the selected leads and drug is tabulated in Table 8.

**TABLE 8:** MM-GBSA results of the selected JAK1 lead compounds and the drug.

S. no.	Molecule ID	∆G _Bind	∆G_Bind_Coulomb	∆G_Bind_Covalent	∆G_Bind_Lipo	∆G_Bind_vdW
1	T6649932	−46.430	−10.816	6.512	−15.475	−55.026
2	ST088474	−44.915	−29.704	5.740	−8.571	−36.645
3	T5923555	−45.936	−11.512	13.940	−15.199	−45.837
4	T5923531	−41.698	−14.070	4.485	−11.368	−47.637
5	T6763842	−43.157	−6.535	5.179	−15.922	−48.815
6	Ruxolitinib	−46.184	−18.883	1.186	−13.853	−38.185

#### Induced Fit Docking Analysis

The accuracy of glide scoring function in identifying the leads was checked using the IFD method. The IFD score was greater than −594, and their corresponding docking score was greater than −8.5. The IFD scores of four lead compounds were higher compared to ruxolitinib (−595.395), whereas the docking scores of four lead compounds were little lower compared with ruxolitinib (−9.725). The highest IFD score and XP score were observed for T6763842 and T5923531. The Electro and Lipophilic Evdw scores of the lead compounds and drugs showed higher lipophilicity compared with electrostatic interactions which implies the important role of lipophilicity in inhibitory activity. The glide XP score, IFD score, lipophilic evdw, electro, and H-bond interaction of the selected leads are given in Table 9. The representation of docked lead compounds and drug present inside the ATP-binding site of JAK1 after induced fit docking is shown in Supplementary Figure S3. The IFD results also confirmed that the selected lead compounds have occupied the ATP-binding site of JAK1 irrespective of receptor flexibility.

**TABLE 9:** Induced fit docking results of the selected JAK1 lead compounds and the drug.

S. no.	Molecule ID	XP score	IFD score	Lipophilic EvdW	Electro	H-bond interaction
1	T6649932	−8.557	−598.316	−5.175	−0.353	Leu959, Glu883
2	ST088474	−8.791	−594.272	−2.492	−0.519	Leu959, Glu957, Arg1007
3	T5923555	−9.343	−598.889	−4.142	−0.638	Leu959, Glu957, Ser963
4	T5923531	−9.941	−598.697	−4.919	−0.701	Leu959, Glu883
5	T6763842	−9.550	−599.541	−5.590	−0.236	Ser963
6	Ruxolitinib	−9.725	−595.395	−3.654	−1.055	Leu959, Glu957

#### Cross-Docking Analysis

The cross-docking results of selected leads indicate that their docking score was greater than −10.00 with JAK1, whereas with respect to other JAKs, their docking score ranges from −5.2 to −8.9. For ruxolitinib, docking scores were −9.178 with JAK1, −9.091 with JAK2, and −10.209 with JAK3. Therefore, the selected lead compounds have shown good selectivity in terms of docking score compared with ruxolitinib. The important components to determine the selectivity of the lead compounds were the electrostatic and hydrophilic components of the docking score. The drug and lead compounds showed higher lipophilicity compared with electrostatic interactions which implies that lipophilicity plays an important role in dictating the selectivity of these molecules. The cross-docking results of the drug and selected leads are given in Table 10. The cross-docking results of ruxolitinib showed higher binding affinity with JAK3 compared with JAK1 and JAK2 indicating its lesser selectivity, whereas the selected lead compounds showed greater affinity and selectivity toward JAK1 compared with other JAK subtypes. Hence, the cross-docking results indicate the selected lead compounds are more selective than the drug.

**TABLE 10:** Cross-docking results of the selected JAK1 lead compounds and the drug.

S. no.	Molecule ID	Glide XP Gscore			Glide energy (Kcal/mol)		
		JAK1	JAK2	JAK3	JAK1	JAK2	JAK3
1	T6649932	−10.623	−6.065	−8.192	−65.073	−59.156	−57.574
2	ST088474	−10.784	−8.938	−7.963	−52.980	−44.112	−40.231
3	T5923555	−10.650	−7.314	−8.298	−56.889	−54.956	−54.964
4	T5923531	−10.608	−7.810	−8.344	−59.950	−57.629	−55.855
5	T6763842	−10.652	−5.243	−8.754	−51.761	−48.136	−48.034
6	Ruxolitinib	−9.178	−9.091	−10.209	−49.475	−48.884	−48.434

### Molecular Dynamics Simulation

#### RMSD Plot Analysis

**FIGURE 5:**
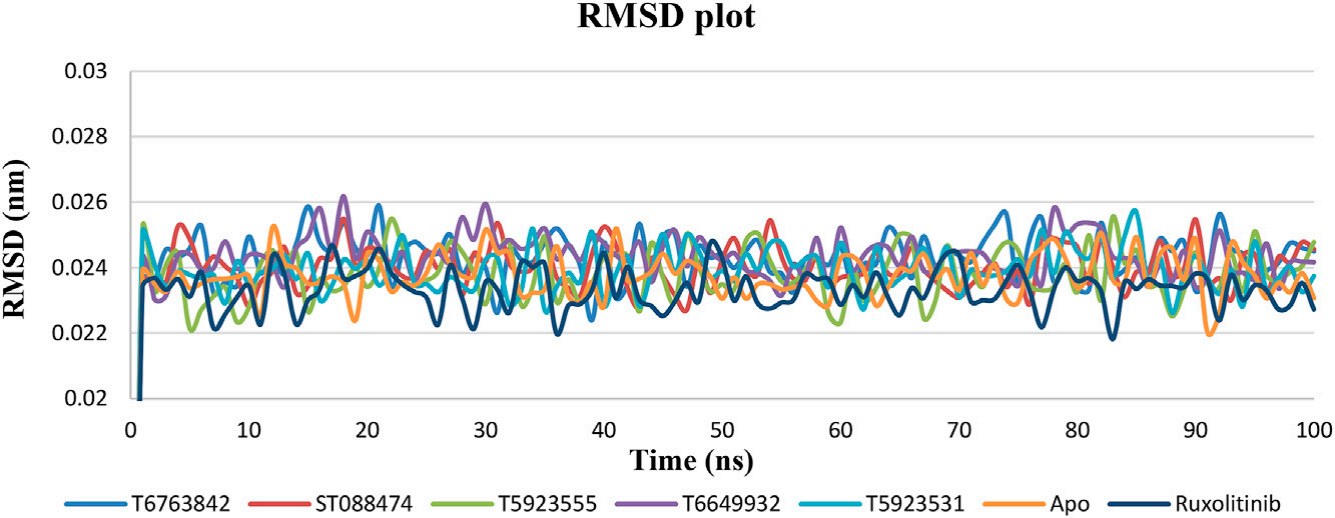
The change in RMSD values of the backbone Cα atoms of JAK1 systems over a period of 100 ns after binding with the lead compounds and drug.

RMSD relative to the respective initial conformations was monitored and analyzed to examine the stability and equilibration of all systems. The RMSD value for both the lead compounds and drug was calculated for the 100ns time scale using the apo form of JAK1 (3EYG) as reference. In Figure 5, it was observed that the RMSD values of both the drug and lead complexes were stable throughout the simulation. Furthermore, RMSD values for all protein backbone atoms attained convergence after 1 ns and maintained a plateau of 0.01 nm after the initial convergence. This suggests that all system simulations reached equilibrium and stabilization during the simulation. RMSD of both drug and lead complexes was found to be in the range of 0.02–0.03 nm indicating the similar stability. The observed smaller RMSD fluctuations for all compounds confirmed that the obtained binding conformations of these lead compounds and drug were highly reasonable.

#### RMSF Plot Analysis

**FIGURE 6:**
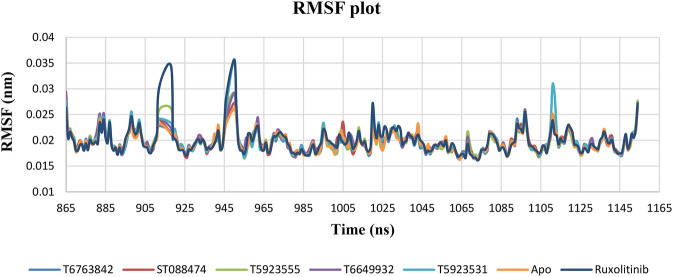
The change in RMSF values of JAK1 residues over a period of 100 ns after binding with the lead compounds and drug.

RMSF of backbone atoms were monitored to identify the strong binding interactions and exemplify the pliability of these lead complexes in the ATP-binding site of JAK1. The RMSF plot shown in Figure 6 indicates very minimal fluctuations were observed during the simulation except the terminal and loop regions of the protein. Most of the fluctuations were between 0.016 and 0.035 nm indicating the stability of the simulated system. Very minimal fluctuations were observed in the residues Pro912, His918, Glu946, Asn950, and Gly951 for all lead compounds and ruxolitinib. The JAK1 ATP-binding site residues that are crucial for binding and fixing the inhibitors have displayed insignificant fluctuations during the course of simulation. However, the most important and selective amino acid residues Leu959 and Glu957 that are important for inhibitor binding attained a quite stable behavior.

#### Rg Plot Analysis

**FIGURE 7:**
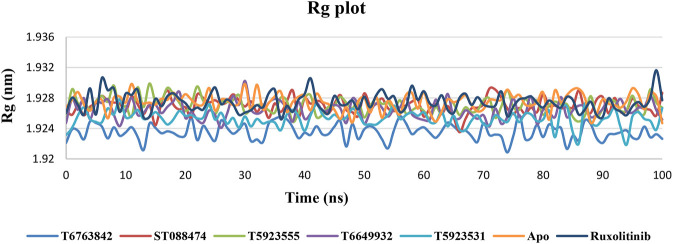
The change in Rg values over a period of 100 ns after binding with the lead compounds and drug.

The level of compactness in the structure of protein due to the presence or absence of ligands was calculated using radius of gyration (Rg) plot (Lobanov et al., 2008). It can be observed that all lead complexes and the drug showed consistently lower Rg values and exhibited a relatively similar nature of compactness in Figure 7. Thus, a relatively consistent Rg value indicates that a stably folded structure was observed throughout the MD simulation.

#### Solvent Accessible Surface Area Plot Analysis

**FIGURE 8:**
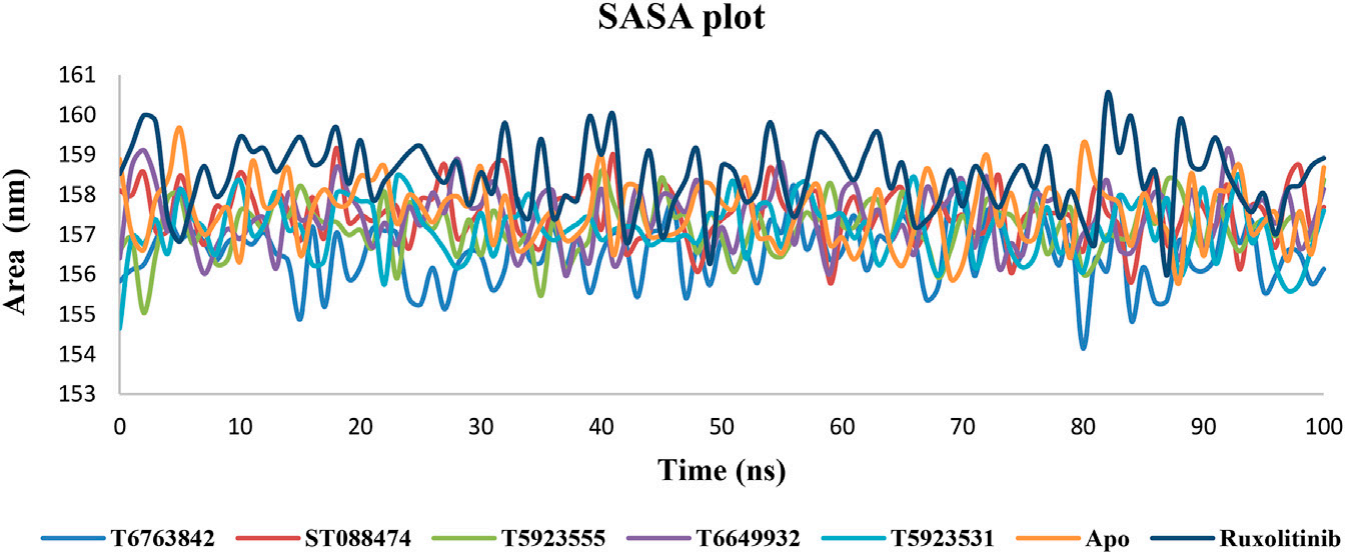
The change in SASA values over a period of 100 ns after binding with the lead compounds and drug.

The solvent accessible surface area (SASA) calculation of the protein–ligand complexes was used for predicting the extent of the conformational changes that occurred during the interaction. The SASA plot shown in Figure 8 indicates that no significant changes in the protein structure were caused by these lead compounds and drug during simulation. Hence, the protein–ligand complexes are relatively stable throughout the simulation.

#### Protein–Ligand Interaction Analysis

**FIGURE 9:**
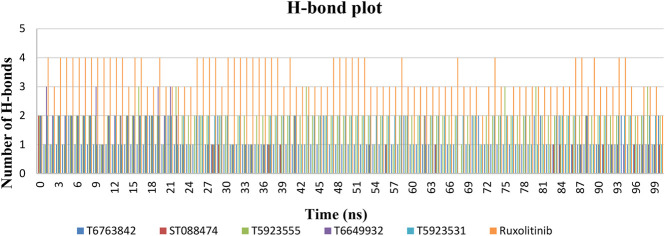
The number of hydrogen bonds formed by lead compounds and drug over the simulation time.

The most significant part in MD simulations is the analysis of protein–ligand interactions because it illustrates the changes in the binding mode of the ligands during simulations. Figure 9 shows the number of H-bond formations over the trajectory for lead compounds and the drug. The H-bonds were the principal binding forces between protein and ligand. The drug ruxolitinib has produced 2–4 H-bonds, whereas lead compounds have produced 0–2 H-bonds throughout the simulation. T5923555, T5923531, and T6763842 have produced 1–3 H-bonds, whereas ST088474 produced one H-bond with JAK1 all through the simulation. Ruxolitinib, T6763842, and T5923555 had retained two H-bonds, and T5923531 had retained one H-bond, whereas T6649932 does not have an H-bond at the end of the simulation. A strong hydrogen bond network was formed mainly by the residues Glu957 and Leu959. T5923555 and T5923531 retained hydrogen bonds with Leu959 and Glu957 at the end of simulation. Moreover, the ATP-binding site residues were almost hydrophobic, which can form strong nonpolar interactions with lead compounds. The detailed protein–ligand interaction residues before and after molecular dynamics simulation (Saddala and Adi 2018) were studied and are given in Table 11. T5923555 and T5923531 were found to be more stable and reliable before and after simulation, and their important interaction (Glu957 and Leu959) remains unchanged throughout the simulation.

**TABLE 11:** The protein–ligand interaction analysis of the selected JAK1 lead compounds and the drug before, during, and after MD simulation.

S. no.	Molecule ID	H-bond interaction					
		Before simulation	During simulation				After simulation
			25 ns	50 ns	75 ns	99 ns	
1	T6649932	Leu959, Glu957, Arg1007	-	Leu959	-	Leu959	-
2	ST088474	Leu959, Glu957, Leu881, Ser963, Glu966	Asp1021	Arg1007, Val1009	Arg1007	Asp1021	Asp1021
3	T5923555	Leu959, Glu957, Arg1007	Leu959, Glu957	Leu959, Glu957	Leu959, Glu957, Leu881	Leu959, Glu957	Leu959, Glu957
4	T5923531	Leu959, Glu957, Arg1007	Leu959, Glu957	Leu959, Glu957	Leu959, Glu957	Leu959, Glu957	Leu959, Glu957
5	T6763842	Leu959, Glu957	Ser963	Ser963	Ser963	Ser963	Ser963
6	Ruxolitinib	Leu959, Glu957	Leu959, Glu957, Leu881	Leu959, Glu957, Leu881	Leu959, Glu957	Leu959, Glu957, Arg1007	Leu959, Glu957

**FIGURE 10:**
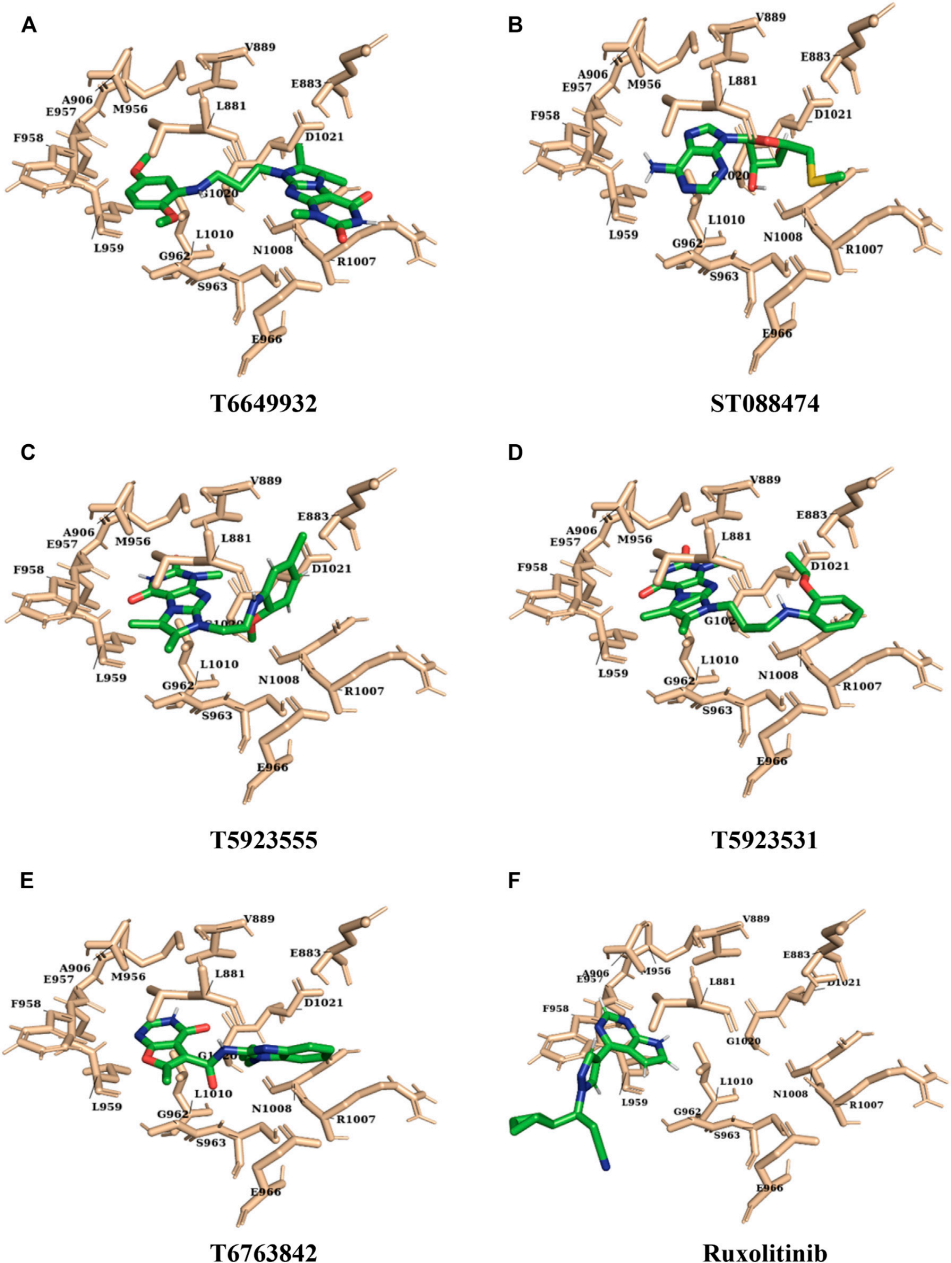
The representation of final conformation of the docked lead compounds and drug (**(A)** T6649932, **(B)** ST088474, **(C)** T5923555, **(D)** T5923531, **(E)** T6763842, and **(F)** ruxolitinib) present inside the ATP-binding site of JAK after molecular dynamics simulation.

The binding mode of the drug and lead compounds after simulation is represented in Figures 10A–F. It was inferred that the initial docked conformation and the final conformation of the lead compounds and drug lie in the same binding pocket (Supplementary Figure S4). Hence, the conformation of the lead compounds was stable inside the binding pocket which, in turn, validates the reliability of the docking results. Furthermore, these absolute results suggest that the identified lead compounds are highly selective and potent and they can be taken for *in vitro* and *in vivo* studies.

### MM-PBSA Calculation

The average binding energy of all the simulated complexes was calculated using the g_mmpbsa tool. The van der Waals energy, electrostatic energy, polar solvation energy, solvent-accessible surface area (SASA) energy, and binding energy were calculated and are tabulated in Table 12. T5923555 and T5923531 have shown good binding energy and van der Waals energy compared with other compounds.

**TABLE 12:** MM-PBSA results obtained from the molecular dynamics trajectory for the selected JAK1 lead compounds and the drug.

S. no.	Molecule ID	van der Waals energy (kJ/mol)	Electrostatic energy (kJ/mol)	Polar solvation energy (kJ/mol)	SASA energy (kJ/mol)	Binding energy (kJ/mol)
1	T6649932	−194.226±16.777	−74.970±32.984	203.123±32.793	−18.207±1.508	−24.281±30.279
2	ST088474	−135.299±6.176	−19.370±4.198	97.066±9.529	−14.916±0.839	−22.519±9.921
3	T5923555	-212.143±8.554	-2.460±7.922	256.402±9.826	-19.217±0.649	-42.581±11.158
4	T5923531	−214.550±9.829	−13.931±5.705	219.798±14.381	−20.106±0.900	−38.790±15.145
5	T6763842	−163.284±11.976	−89.322±11.300	230.810±17.465	−15.839±0.839	−27.636±15.186
6	Ruxolitinib	−185.994±50.799	−34.821±65.738	206.801±108.464	−17.040±6.025	−24.054±36.312

### Density Functional Theory Calculation

Molecular descriptors based on the electron density of the molecules were studied using Gaussian. Based on HOMO energy (E_HOMO_) and LUMO energy (E_LUMO_), descriptors such as ΔE, η, σ, μ, χ, and ω were calculated. The smaller energy gap (ΔE) for all lead compounds suggests that they can easily transit from HOMO to LUMO, which is important for the molecular reactivity. Since the decrease in electronegativity (χ) value is proportional to the increase in inhibitive efficiency (Zhan et al., 2003), these leads would have higher inhibitory activity because of their lower electronegativity value. The statistical values of the calculated molecular descriptors are tabulated in Table 13. The smaller energy gap, lower electronegativity, and higher dipole moment that are vital for the inhibitory effect of a molecule were observed which validates the better inhibitory activity for the selected lead compounds.

**TABLE 13:** The statistical results of the DFT-based descriptors for the selected lead compounds and the drug.

S. no.	Total energy (a.u.)	Energy of		ΔE	Dipole moment (debye)	η	σ	*χ*	*μ*	ω
		εHOMO (Kcal/mol)	εLUMO (Kcal/mol)							
1	−1,195.61	−0.20	−0.04	4.48	3.06	2.24	0.22	−3.20	3.20	2.29
2	−1,325.80	−0.21	−0.01	5.43	3.48	2.71	0.18	−3.08	3.08	1.75
3	−1,675.02	−0.29	−0.07	6.09	5.59	3.04	0.16	−4.83	4.83	3.84
4	−1824.84	−0.22	−0.08	3.84	8.02	1.92	0.26	−4.08	4.08	4.33
5	−1,695.05	−0.25	−0.07	4.97	5.14	2.48	0.20	−4.28	4.28	3.69
R	−987.14	−0.22	−0.05	4.59	4.09	2.29	0.22	−3.64	3.64	2.88

T6649932 (1), ST088474 (2), T5923555 (3), T5923531 (4), T6763842 (5), ruxolitinib (R). ΔE, band energy gap (εLUMO-εHOMO); η, absolute hardness; σ, global softness; χ, electronegativity; μ, chemical potential; ω, electrophilicity index.

## Conclusion

Pharmacophore modeling, virtual screening, and molecular docking are the rational methods for the identification of novel leads with diverse chemical scaffold. Therefore, ligand-based pharmacophore modeling combined with virtual screening and docking was applied in this study to discover novel, potent, and selective virtual hits for JAK1 enzyme. Initially, the ligand-based pharmacophore models were generated and validated using the potency and selectivity validation methods. Eight pharmacophore models were selected and taken for pharmacophore-based virtual screening against six databases. The hits obtained from screening were filtered through ADME prediction and molecular docking. The binding free-energy calculation and induced fit docking methods were employed to validate the docking results. Subsequently, cross docking was carried out to identify the lead compounds that are more selective toward JAK1. Finally, the top five lead compounds were selected and taken for molecular dynamics and the DFT study. Among the five compounds, T5923555 and T5923531 were found to be the best leads and can be further validated using *in vitro* and *in vivo* methods.

## Data Availability

The original contributions presented in the study are included in the article/Supplementary Material, further inquiries can be directed to the corresponding authors.
