# Supplementary material for: Identification of Potent and Selective JAK1 Lead Compounds Through Ligand-Based Drug Design Approaches
Source: Front Pharmacol. 2022 Apr 21;13:837369. doi: 10.3389/fphar.2022.837369 (PMC9068899; doi:10.3389/fphar.2022.837369)
Supplement: Supplementary file 1 [file DataSheet1.pdf]

Supplementary Table 1: The selected JAK1 hits from molecular docking study.

S. No	Hypothesis	Molecule ID	Fitness	Docking Score SP	Docking Score XP	Glide Energy
1	ADHRRR	T5920852	1.55	-6.402	-11.510	-54.576
2	ADPRR	T5925648	1.90	-7.902	-11.162	-50.357
3	DDHRR	ZINC41165444	2.05	-8.509	-11.097	-45.811
4	ADPRR	ZINC95449166	2.07	-6.683	-10.894	-50.271
5	ADPRR	ZINC65591421	2.04	-5.764	-10.883	-46.471
6	ADPRR	F3289-1086	2.09	-7.945	-10.867	-57.806
7	DDHRR	T6763842	1.83	-7.550	-10.671	-51.703
8	DDHRR	ST088474	1.73	-9.293	-10.653	-50.800
9	DDHRR	T6229048	1.87	-8.456	-10.610	-51.663
10	ADPRR	T6112362	1.84	-8.158	-10.532	-58.182
11	DDHRR	ZINC97480470	2.02	-10.712	-10.463	-57.656
12	ADPRR	T5760493	1.85	-7.388	-10.385	-48.594
13	DDRRR	T6649932	1.55	-7.536	-10.335	-61.771
14	ADPRR	ZINC72148730	2.02	-7.570	-10.327	-47.485
15	ADRRR	ZINC77955167	2.38	-6.693	-10.322	-53.710
16	DDHRR	ZINC72405250	2.15	-9.124	-10.306	-42.617
17	DDRRR	T5923531	1.52	-7.102	-10.303	-57.350
18	DHRRR	ZINC68605539	2.00	-7.213	-10.274	-45.465
19	DPRRR	ZINC77955168	2.06	-5.524	-10.266	-49.226
20	DDHRR	ZINC97480473	2.03	-10.146	-10.260	-60.729
21	ADPRR	ZINC65611240	2.04	-7.597	-10.238	-45.553
22	DDHRR	ZINC78525289	2.19	-8.123	-10.230	-56.090
23	ADPRR	ZINC19741573	2.11	-6.925	-10.221	-42.384
24	ADPRR	T6900679	1.82	-6.196	-10.206	-49.888
25	ADRRR	ZINC77955172	2.13	-5.963	-10.201	-20.930
26	ADPRR	ZINC97159261	2.03	-7.367	-10.185	-50.345
27	ADPRR	ZINC97160064	2.01	-8.715	-10.182	-46.672
28	DHRRR	ZINC01545892	2.00	-6.951	-10.137	-45.520
29	ADPRR	T5919457	1.84	-6.536	-10.123	-51.724
30	ADPRR	T5773547	1.84	-6.877	-10.114	-57.905
31	DDHRR	ZINC05903136	2.11	-8.228	-10.111	-44.202
32	DDHRR	F2491-0101	2.12	-7.766	-10.105	-45.273
33	ADPRR	T5661069	1.87	-6.811	-10.080	-53.208
34	ADPRR	ZINC73737252	2.08	-7.180	-10.072	-46.391
35	ADPRR	T6640882	1.83	-5.524	-10.071	-46.301
36	ADPRR	G275-1407	1.91	-7.003	-10.066	-48.996
37	DDHRR	ZINC72171525	2.22	-8.156	-10.062	-46.311

S. No	Hypothesis	Molecule ID	Fitness	Docking Score SP	Docking Score XP	Glide Energy
38	ADPRR	T6385529	1.92	-7.210	-10.060	-48.558
39	ADPRR	ZINC95449165	2.02	-5.933	-10.059	-51.392
40	ADPRR	ZINC95469612	2.06	-9.074	-10.052	-51.190
41	DDHRR	T6789630	1.72	-8.085	-10.037	-58.596
42	DDRRR	ZINC77971158	2.37	-7.943	-10.037	-50.510
43	ADPRR	ZINC71839171	2.01	-7.376	-10.021	-49.216
44	ADPRR	T6865686	1.92	-6.340	-10.020	-52.972
45	DDRRR	T5923555	1.77	-8.482	-10.015	-57.500
46	ADPRR	T6466231	1.84	-6.162	-10.012	-56.528
47	DDHRR	ZINC50767136	2.11	-8.849	-10.012	-47.800
48	ADPRR	T5923533	2.01	-6.635	-10.009	-59.962
49	DDHRR	T6076538	1.71	-6.545	-9.987	-54.848
50	DDRRR	T5923502	1.72	-7.674	-9.986	-56.441
51	DDRRR	ZINC77971142	2.25	-8.643	-9.957	-53.041
52	DDRRR	ZINC77971114	2.24	-8.639	-9.953	-53.005
53	DHRRR	T5923452	1.84	-5.751	-9.939	-55.519
54	DPRRR	ZINC13541700	2.07	-8.792	-9.930	-52.928
55	DDRRR	T5881519	1.51	-6.373	-9.882	-47.103
56	DDRRR	ZINC77971139	2.27	-8.553	-9.872	-50.493
57	DDRRR	T5923554	1.56	-7.265	-9.866	-57.223
58	ADRRR	T05034595	1.72	-5.378	-9.852	-42.346
59	DPRRR	F3234-0530	2.05	-9.017	-9.841	-52.467
60	DDRRR	T5927095	1.78	-7.570	-9.837	-56.367
61	DHRRR	ZINC77955184	2.32	-6.045	-9.833	-50.798
62	DDRRR	T5927094	1.56	-7.686	-9.827	-56.589
63	DHRRR	T5707836	2.17	-7.900	-9.827	-51.760
64	ADRRR	T5865427	1.80	-8.327	-9.826	-51.927
65	DDHRR	T6052252	1.68	-6.692	-9.819	-46.423
66	DDHRR	T5506536	1.77	-4.042	-9.816	-48.785
67	DDHRR	T6783264	1.74	-7.548	-9.813	-52.850
68	DDRRR	F3289-1128	1.57	-5.178	-9.808	-55.279
69	DHRRR	F3259-0214	1.87	-8.507	-9.807	-49.673
70	DDRRR	T5927106	1.83	-7.125	-9.803	-56.420
71	DHRRR	T5769827	1.85	-6.422	-9.803	-61.820
72	DDRRR	T5923480	1.78	-6.759	-9.797	-57.541
73	DDRRR	T5927092	1.50	-6.235	-9.794	-58.554
74	DDHRR	F6000-0016	1.75	-8.034	-9.793	-56.378
75	DDRRR	T6649944	1.82	-8.808	-9.771	-54.217

S. No	Hypothesis	Molecule ID	Fitness	Docking Score SP	Docking Score XP	Glide Energy
76	DDHRR	T5564236	1.79	-7.526	-9.762	-54.207
77	DDRRR	T5923496	1.69	-7.299	-9.754	-54.701
78	DDRRR	T5927123	1.54	-5.955	-9.739	-56.169
79	DHRRR	T5720930	1.81	-8.840	-9.729	-48.047
80	DDRRR	F3289-0901	1.81	-7.839	-9.727	-54.174
81	DDRRR	T5927134	1.56	-7.465	-9.719	-51.019
82	DDRRR	F3289-1111	1.59	-7.191	-9.711	-55.262
83	DDHRR	T5858364	1.69	-8.797	-9.710	-49.045
84	DHRRR	T5648531	1.72	-7.520	-9.707	-56.272
85	DPRRR	F3234-0076	2.06	-8.958	-9.703	-50.851
86	DDHRR	T6215014	1.74	-7.148	-9.703	-51.314
87	DDRRR	T6652943	1.68	-7.230	-9.690	-46.975
88	DDRRR	T5923437	1.84	-6.298	-9.684	-57.616
89	DHRRR	T5941474	1.79	-7.673	-9.648	-56.128
90	DHRRR	T5740560	1.82	-7.723	-9.630	-56.340

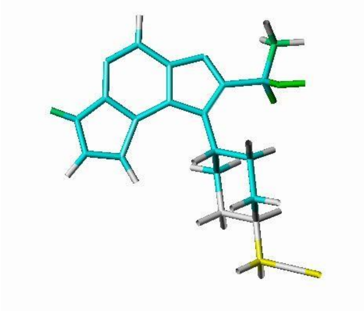

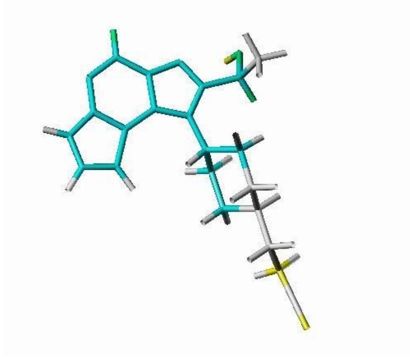

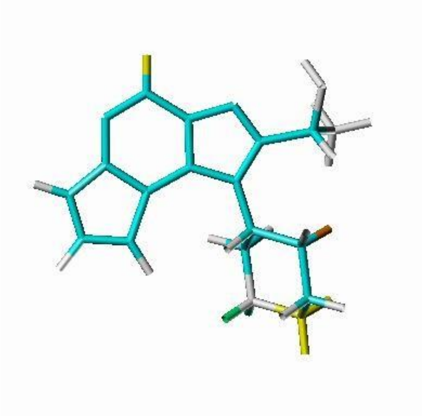

Compound 51 **Compound 52** **Compound 26**

Supplementary Figure 1: The contribution maps of highly active compounds in the dataset.

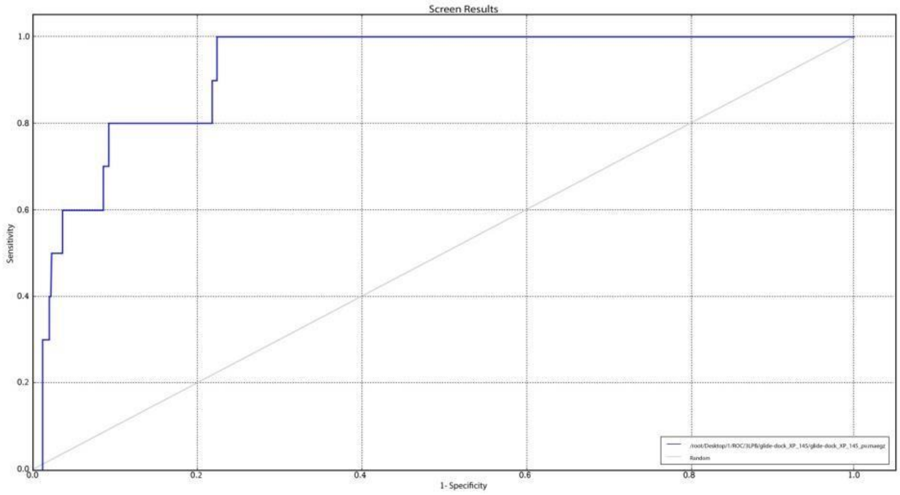

Supplementary Figure 2: The validation of Glide XP docking program using ROC plot.

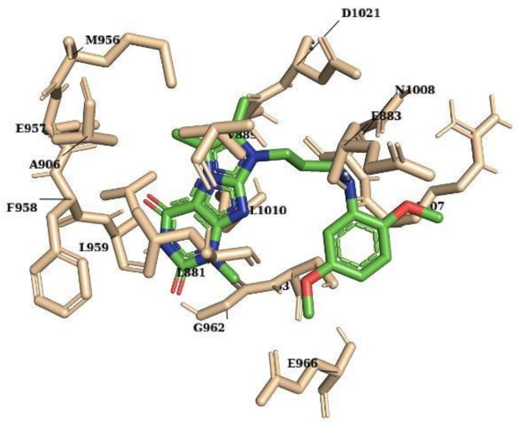

(a) **T6649932**

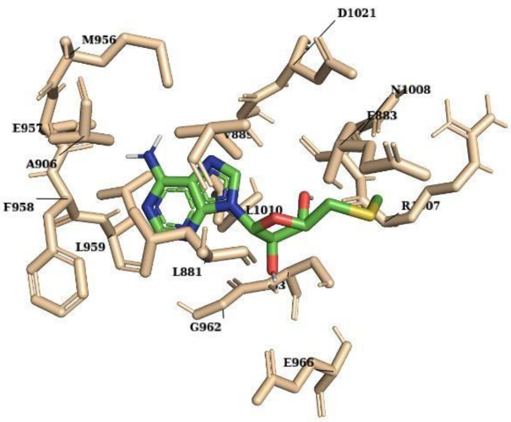

(b) **ST088474**

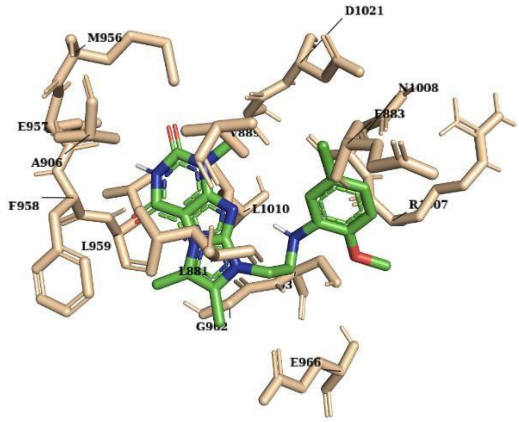

(c) **T5923555**

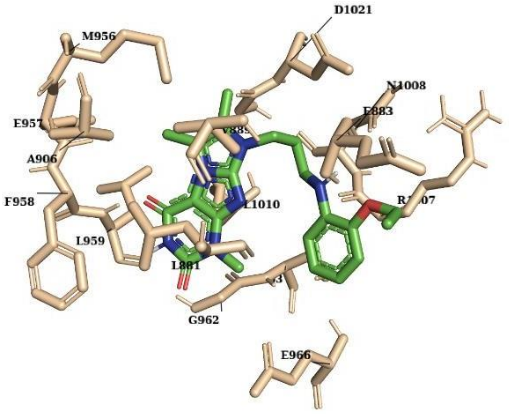

(d) **T5923531**

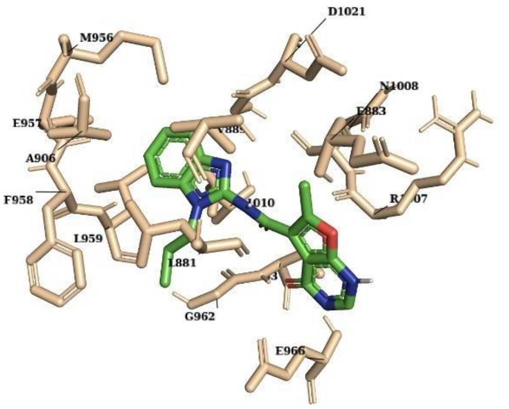

(e) **T6763842**

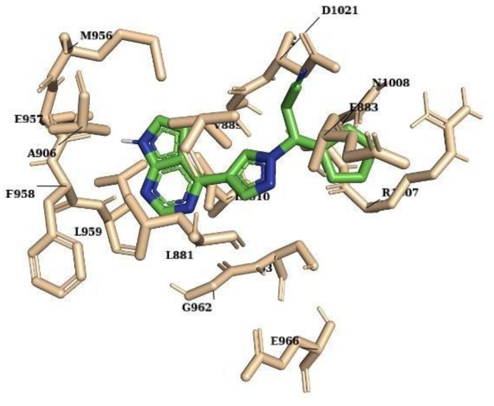

(f) **Ruxolitinib**

Supplementary Figure 3 (a)-(f): The representation of docked lead compounds and drug present inside the ATP-binding site of JAK1 after induced fit docking.

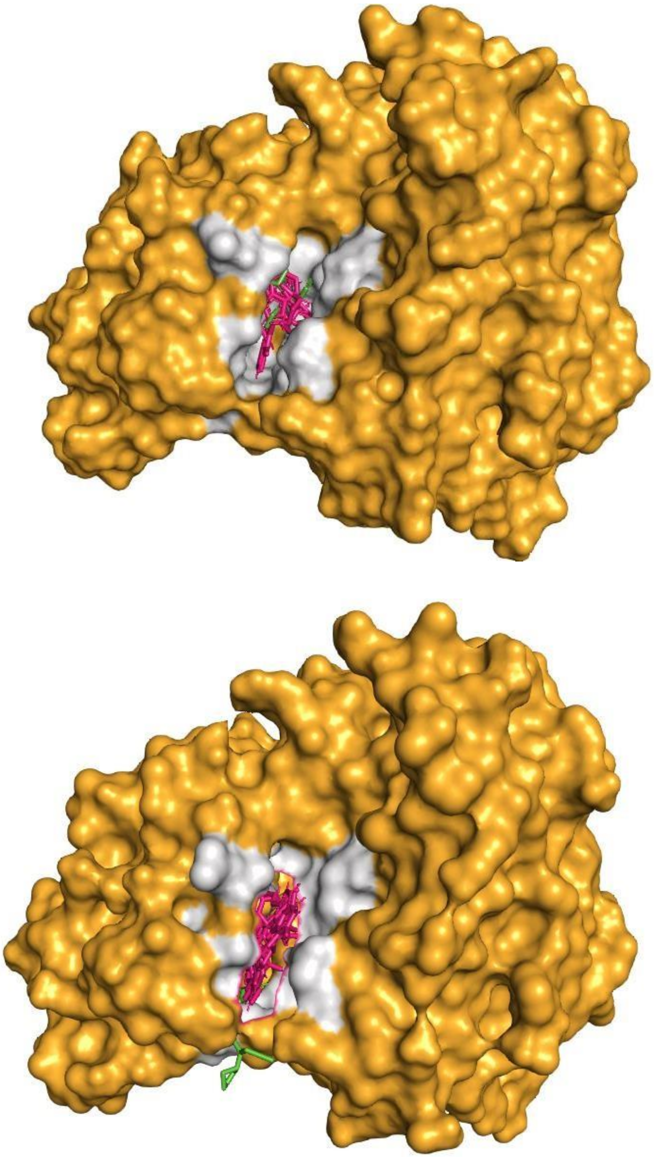

Supplementary Figure 4: The representation of lead compounds (pink) and drug (green) inside the ATP-binding site after molecular docking and dynamics study.
